# Infinite-body optimal transport with Coulomb cost

**DOI:** 10.1007/s00526-014-0803-0

**Published:** 2014-12-17

**Authors:** Codina Cotar, Gero Friesecke, Brendan Pass

**Affiliations:** 1 Department of Statistical Science, University College London, London, UK; 2 Department of Mathematics, Technische Universität München, Munich, Germany; 3 Department of Mathematical and Statistical Sciences, University of Alberta, Edmonton, Canada

**Keywords:** N-representability, Density functional theory, Hohenberg-Kohn functional, N-body optimal transport, Infinite-body optimal transport, Coulomb cost, Exchange-correlation functional, de Finetti’s Theorem, Finite exchangeability, N-extendability, 49S05, 65K99, 81V55, 82B05, 82C70, 92E99, 35Q40

## Abstract

We introduce and analyze symmetric infinite-body optimal transport (OT) problems with cost function of pair potential form. We show that for a natural class of such costs, the optimizer is given by the independent product measure all of whose factors are given by the one-body marginal. This is in striking contrast to standard finite-body OT problems, in which the optimizers are typically highly correlated, as well as to infinite-body OT problems with Gangbo–Swiech cost. Moreover, by adapting a construction from the study of exchangeable processes in probability theory, we prove that the corresponding $N$-body OT problem is well approximated by the infinite-body problem. To our class belongs the Coulomb cost which arises in many-electron quantum mechanics. The optimal cost of the Coulombic N-body OT problem as a function of the one-body marginal density is known in the physics and quantum chemistry literature under the name *SCE functional*, and arises naturally as the semiclassical limit of the celebrated Hohenberg-Kohn functional. Our results imply that in the inhomogeneous high-density limit (i.e. $N\rightarrow \infty$ with arbitrary fixed inhomogeneity profile $\rho {/}N$), the SCE functional converges to the mean field functional. We also present reformulations of the infinite-body and N-body OT problems as two-body OT problems with representability constraints.

## Introduction

### Semi-classical electron-electron interaction functional and connection to optimal transport

This work is motivated by, and contributes to, the longstanding quest in physics, chemistry and mathematics to design and justify approximations to the energy functional of many-electron quantum mechanics in terms of the one-body density.

A simplified yet still formidable challenge consists in understanding the following “semi-classical” interaction energy functional obtained by a constrained search over $N$-point densities with given one-body density $\rho$. This functional, introduced in the physics literature by Seidl et al. [61–63], is given by

1.1 $$\begin{aligned} V_{ee}^{SCE}[\rho ] := \inf _{\gamma _N\in {\mathcal {P}}^N_{sym}(\mathbb {R}^{3}),\,\gamma _N\mapsto \rho /N} C_N[\gamma _N], \end{aligned}$$

where $\rho$ is a given nonnegative function on $\mathbb {R}^3$ with $\int _{\mathbb {R}^3} \rho = N$ (physically: the total electron density of an atom or molecule with $N$ electrons) and

1.2 $$\begin{aligned} C_N[\gamma _N] := \int _{\mathbb {R}^{3N}} \sum _{1\le i<j\le N} \frac{1}{|x_i-x_j|} d\gamma _N(x_1,\ldots ,x_N). \end{aligned}$$

Here ${\mathcal {P}}_{sym}^{N}(\mathbb {R}^3)$ is the space of probability measures $\gamma _N$ on $\mathbb {R}^{3N}$ which satisfy the symmetry condition

1.3 $$\begin{aligned} \gamma _N(A_1\times \cdots \times A_N)&= \gamma _N(A_{\sigma (1)}\times \cdots \times A_{\sigma (N)}) \text{ for } \text{ all } \text{ Borel } \text{ sets }~A_1,\ldots ,A_N\subseteq \mathbb {R}^3 \nonumber \\&\quad \text{ and } \text{ all } \text{ permutations } \sigma , \end{aligned}$$

and the notation $\gamma _N\mapsto \rho /N$ means that $\gamma _N$ has one-body density $\rho$ (physics terminology) or equivalently equal $\mathbb {R}^3$-marginals $\rho /N$ (probability terminology),

1.4 $$\begin{aligned} \gamma _N(\mathbb {R}^{3(i-1)}\times A_i \times \mathbb {R}^{3(N-(i-1))}) = \int _{A_i}\frac{\rho (x)}{N}\,dx \text{ for } \text{ all } A_i\subseteq \mathbb {R}^3 \text{ and } \text{ all } i=1,\ldots ,N.\nonumber \\ \end{aligned}$$

The normalization factor $1/N$ in (1.1) and (1.4) is owed to the convention in many-electron quantum mechanics that the one-body density $\rho$ should integrate to the number of particles in the system, i.e. $\int _{\mathbb {R}^3} \rho = N$, whereas the marginal density in the sense of probability theory, denoted in the sequel by $\mu$, should integrate to $1$. The functional (1.1) is commonly called the *SCE functional*, where the acronym SCE stands for strictly correlated electrons; the fact that e.g. for $N=2$, minimizers concentrate on lower-dimensional sets of form $x_2=T(x_1)$ (see (1.5) below) has the physical interpretation that given the position of the first electron, the position of the second electron is strictly determined. The connection of the functional (1.1) with many-electron quantum mechanics which motivated this work is explained at the end of this Introduction.

We remark that dropping the symmetry requirement on $\gamma _N$ would not alter the minimum value in (1.1), since the functional $C_N$ takes the same value on a nonsymmetric measure as on its symmetrization.

Because of the appearance of the $N$-particle configurations $(x_1,\ldots ,x_N)$ and of the $N$-body cost $\sum _{i<j}1/{|x_i-x_j|}$ in $C_N[\gamma _N]$, we call this functional an $N$-*body mass transportation functional* or *an optimal transport problem with* $N$ *marginals*, and the problem (1.1) of minimizing it an $N$-*body optimal transport problem*. The functional $V_{ee}^{SCE,N}$ can be interpreted as the *minimum cost of an optimal transport problem as a functional of the marginal measure*. In the case $N=2$, one is dealing with a standard (two-body or two-marginal) optimal transport problem of form

$$\begin{aligned} \text{ Minimize } \int _{\mathbb {R}^{2d}} c(x_1,x_2) d\gamma _2(x_1,x_2) \text{ over } \gamma _2\in {\mathcal {P}}(\mathbb {R}^{2d})~ \\ \text{ subject } \text{ to } \gamma _2(A\times \mathbb {R}^3)=\gamma _2(\mathbb {R}^3\times A)=\mu (A) \quad \text{ for } \text{ all } A\subseteq \mathbb {R}^3, \end{aligned}$$

where $c\, : \, \mathbb {R}^d\times \mathbb {R}^d\rightarrow \mathbb {R}\cup \{\infty \}$ is a *cost function* and ${\mathcal {P}}(\mathbb {R}^{2d})$ is the space of probability measures on $\mathbb {R}^{2d}$.

### Previous results

It was not realized until recently [10, 17] that the minimization problem in (1.1) has the form of an optimal transport problem and can, especially in the case $N=2$, be fruitfully analyzed via methods from OT theory.

OT problems with two marginals have been studied extensively in the mathematical literature for a large variety of cost functions; see, for example [9, 31] for some influential results in the area and [65] for a comprehensive treatment. A central insight in this setting is that, under fairly weak conditions on the cost function and marginals, the optimal measure is unique and of Monge type, i.e. it concentrates on the graph of a map over $x_1$. That is to say,

1.5 $$\begin{aligned} \gamma _2&= (I\times T)_\sharp \mu \text{(OT } \text{ notation) } \text{ or } \text{ equivalently } \gamma _2(x,y)=\mu (x)\delta _{T(x)}(y) \nonumber \\&\quad \text{(physics } \text{ notation) } \text{ for } \text{ some } \text{ map } T\, : \, \mathbb {R}^d\rightarrow \mathbb {R}^d. \end{aligned}$$

Even though the Coulomb cost lies outside the costs treated in standard OT theory (where positive power costs like $|x-y|$ or $|x-y|^2$ are prototypical), the result (1.5) has recently been extended to the 2-body OT problem with Coulomb cost, (1.1) with $N=2$ [10, 17], confirming earlier nonrigorous results in the physics literature [61, 62].

Much less is known about $N$-body OT problems with $N\ge 3$. Here the OT literature has focused on special cost functions [10, 13, 15, 16, 18, 27, 32, 35, 38, 42, 49–51, 54, 59, 60] and the structure of solutions is highly dependent on the cost function. For certain costs, solutions concentrate on graphs over the first marginal, as in the two body case, while for others the solutions can concentrate on high dimensional submanifolds of the product space. In particular, despite its importance in electronic structure theory, very little is known regarding the structure of the solutions of the $N$-body OT problem with Coulomb cost (1.1). Let us note, however, that the study of Monge–Kantorovich problems with symmetry constraints has been intitiated in [34] and continued in [15, 16, 29, 33, 35], the last two papers dealing with the Coulomb cost.

### Main results

Here we focus on problem (1.1) in the *regime of large* $N$, i.e. the “opposite” regime of the hitherto best understood case $N=2$. We present two main results. The first introduces and analyzes the associated *infinite-body OT problem*. Remarkably, for a natural class of costs which includes the Coulomb cost, the infinite-body problem is uniquely minimized by the independent product measure all of whose factors are given by the one-body marginal. See Definition 1.1 below for the precise statement. This stands in surprising contrast to the pair of recent papers [Pass12a] and [Pass12b]. There costs of Gangbo–Swiech type are analyzed and it is shown that the optimizer is a Monge type solution; that is, any two of the variables are completely dependent rather than completely independent. Our second main result says that the corresponding $N$-body OT problem is well approximated by the infinite-body problem; in particular we show that the optimal cost per particle pair of the $N$-body problem converges to that of the infinite-body problem as $N$ gets large. See Theorem 1.2 for the precise statement.

### Connection with many-electron quantum mechanics and the Hohenberg–Kohn functional

Next let us explain the connection with, and implications for, many-electron quantum mechanics. Heuristically, the functional $V_{ee}^{SCE}$ is the semiclassical limit of the celebrated Hohenberg–Kohn functional [37],

1.6 $$\begin{aligned} V_{ee}^{SCE}[\rho ] = \lim _{\hbar \rightarrow 0} F^{HK}[\rho ], \end{aligned}$$

where

1.7 $$\begin{aligned} F^{HK}[\rho ]:=\min _{\Psi \in {\mathcal {A}}_N, \, \Psi \mapsto \rho } \langle \Psi , ( \hbar ^2 \widehat{T} + \widehat{V}_{ee})\Psi \rangle . \end{aligned}$$

Here $\widehat{T}=-\frac{1}{2}\Delta$, $\Delta$ is the Laplacian on $\mathbb {R}^{3N}$, and the resulting contribution to the functional is the quantum mechanical kinetic energy of the system, $\widehat{V}_{ee}$ is the electron-electron operator which acts by multiplication with the function $V_{ee}(x_1,\ldots ,x_N)=\sum _{1\le i<j\le N}1/|x_i-x_j|$, ${{\mathcal {A}}_N}$ denotes the set of antisymmetric, square-integrable functions $\Psi \, : \, (\mathbb {R}^3\times \mathbb {Z}_2)^N\rightarrow \mathbb {C}$ with square-integrable gradient and $L^2$ norm $1$, $\langle \cdot ,\cdot \rangle$ is the $L^2$ inner product, and the notation $\Psi \mapsto \rho$ means that the associated $N$-point position density

1.8 $$\begin{aligned} \gamma _N(x_1,\ldots ,x_N) = \sum _{s_1,\ldots ,s_N\in \mathbb {Z}_2} |\Psi (x_1,s_1,\ldots ,s_N,x_N)|^2 \end{aligned}$$

satisfies $\gamma _N\mapsto \rho /N$. The class of single-particle densities on which $F^{HK}$ is defined is the image of $\mathcal{A}_N$ under the map $\Psi \mapsto \rho$. By a result of Lieb [Li83], this class equals the set of functions $\rho \, : \, \mathbb {R}^3\rightarrow \mathbb {R}$ which are nonnegative, have integral $N$, and have the property that $\sqrt{\rho }$ belongs to the Sobolev space $H^1(\mathbb {R}^3)$. The HK functional constituted the birth of modern density functional theory (DFT). DFT approximates $F^{HK}$ by simpler yet still remarkably accurate functionals of the one-body density amenable to efficient numerical minimization, and is the currently most widely used method for numerical electronic structure computations for complex systems ranging from condensed matter over surfaces and nanoclusters to large molecules. For further information about the HK functional and mathematical aspects of the challenge to approximate it by computationally simpler functionals we refer to our recent paper [17] and the literature cited therein. A rigorous justification of Eq. (1.7) is given in [17] (for N = 2) and [18] (for an arbitrary number of particles). While the proof itself shall not concern us here, we remark that there is indeed something to prove: minimizers $\gamma _N$ of the limit problem in (1.1) are typically singular measures and hence do not arise as $N$-point densities (1.8) of any quantum wavefunction $\Psi \in {\mathcal {A}}_N$, making it a nontrivial task to construct a wavefunction with precisely the same one-body density as $\gamma _N$ for which the quantum expectation value on the right hand side of (1.7) is well defined and close to the value $V_{ee}^{SCE}[\rho ]=C_N[\gamma _N]$ on the left hand side of (1.6).

Together with the companion result (1.6), Theorem 1.2 says that the Hohenberg–Kohn functional $F^{HK}$ is rigorously asymptotic, in the regime of small $\hbar$, a large number of electrons, and a fixed inhomogeneity profile $\rho /N$, to the mean field functional

1.9 $$\begin{aligned} J[\rho ] = \frac{1}{2} \int _{\mathbb {R}^6} \frac{1}{|x_1-x_2|}\rho (x_1)\rho (x_2)\, dx_1\, dx_2. \end{aligned}$$

See Corollary 1.4 below for the precise statement. This result answers an open question raised by us in [27], where we observed this correspondence for a toy model (one-body densities supported on two points, cost favouring different-site occupancy over same-site occupancy) for which the $N$-body OT problem in (1.1) can be solved explicitly.

### Precise statement of main results

With a view to the application to density functional theory, we will work in the following setting even though some of our main results could be stated and proved for more general spaces, such as Polish spaces for Theorem 1.3.

#### **Definition 1.1**

(Infinite-dimensional Borel $\sigma$-algebra) Let $(\Omega _i^d, \mathcal{{F}}_i^d):=(\mathbb {R}^d, \mathcal{B}(\mathbb {R}^d))$, where $i=1,2,\ldots , N,\ldots ,$ and $d\ge 1$. The underlying $\sigma$-field is the Borel $\sigma$-field. We define the *infinite-dimensional cartesian product* $\Omega _\infty ^d$ by $\Omega _\infty ^d:=\times _{i=1}^\infty \Omega _i^d,$ and the *infinite-dimensional Borel* $\sigma$ *-algebra* $\mathcal{B}_\infty ^d$ as the Borel $\sigma$-algebra generated by the open subsets of $\Omega _\infty ^d$ of the form $\prod _{i=1}^\infty A_i$, $A_i\in \mathcal{B}(\mathbb {R}^d)$, where $A_i=\Omega _i^d$ for all but a finite number of $i$.

For an abstract measurable space $(\mathcal{S}, \mathcal{B}(\mathcal{S}))$, we define similarly $(\mathcal{S}^\infty , \mathcal{B}^\infty (\mathcal{S}))$ as the cartesian product of $(\mathcal{S}, \mathcal{B}(\mathcal{S}))$.

To simplify the notation, we will write $(\mathbb {R}^d)^\infty$ instead of $\Omega _\infty ^d$. Throughout the paper, if $\mu \in {\mathcal {P}}(\mathbb {R}^d)$ has a Lebesgue-integrable density, the latter is also denoted by $\mu$.

For all $N\in \mathbb {N}, N\ge 2$, let the *cost function* $c_N:\underbrace{\mathbb {R}^d\times \cdots \times \mathbb {R}^d}_\mathrm N times \rightarrow {\mathbb {R}}_+\cup \{\infty \}$ be defined by

1.10 $$\begin{aligned} c_N(x_1,\ldots ,x_N):=\sum _{1\le i<j\le N} c(x_i,x_j), \end{aligned}$$

where $c:\mathbb {R}^{2d}\rightarrow [0,\infty ) {\cup } \{\infty \}$ is assumed throughout to be Borel-measurable and symmetric (the latter means that $c(x,y)=c(y,x)$ for all $x,y\in \mathbb {R}^d$). For any $N\in \mathbb {N}$, and any infinite-dimensional probability measure $\gamma$ belonging to the space ${{\mathcal {P}}}^{\infty }_{sym}(\mathbb {R}^d)$ defined below, let

1.11 $$\begin{aligned} {C}_N[\gamma ]&= \int _{(\mathbb {R}^d)^\infty } c_N(x_1\ldots ,x_N)d\gamma (x_1,x_2,\ldots ,x_N,\ldots )\nonumber \\&= \sum _{1\le i<j\le N} \int _{(\mathbb {R}^d)^\infty } c(x_i,x_j)d\gamma (x_1,x_2\ldots ,x_N,\ldots ). \end{aligned}$$

Here the domain of this functional is the space ${\mathcal {P}}_{sym}^\infty (\mathbb {R}^d)$ of symmetric Borel probability measures on $(\mathbb {R}^d)^\infty$. For a more detailed discussion of the notion of infinite-dimensional symmetric Borel measures see for example [22]. Symmetric means that for all $N$ and for all $N$-tuple $(i_1,\ldots ,i_N)$ of indices with $1\le i_1<i_2<\cdots <i_N$,

$$\begin{aligned}&\gamma (\mathbb {R}^{d(i_1-1)}\times A_{i_1}\times \mathbb {R}^{d(i_2-i_1-1)}\times A_{i_2}\times \cdots \times A_{i_N}\times \mathbb {R}^d\times \cdots )\\&\quad = \gamma (\mathbb {R}^{d(i_1-1)}\times A_{\sigma ({i_1})}\times \mathbb {R}^{d(i_2-i_1-1)}\times A_{\sigma ({i_2})}\times \cdots \times A_{\sigma (i_N)}\times \mathbb {R}^d\times \cdots ) , \end{aligned}$$

for all Borel sets $A_{i_1}, A_{i_2},\ldots A_{i_N}\subset \mathbb {R}^d$ and for all permutations $\sigma$ of $\{ i_1,i_2,\ldots , i_N\}$. As $N\rightarrow \infty$, the problem of minimizing $C_N$ subject to the marginal constraint $\gamma \rightarrow \mu$ turns into a meaningful, and—as we shall see—very interesting, limit problem:

1.12 $$\begin{aligned} \text{ Minimize } C_\infty [\gamma ]&:= \lim _{N\rightarrow \infty } \frac{1}{{N\atopwithdelims ()2}} C_N[\gamma ] \text{ over } \text{ infinite-dimensional } \text{ probability } \text{ measures } \nonumber \\&\gamma \in {\mathcal {P}}^\infty _{sym}(\mathbb {R}^d) \text{ with } \gamma \rightarrow \mu . \end{aligned}$$

Here the standard notation $\gamma \mapsto \mu$ means that $\gamma$ has one-body marginal $\mu$, i.e. $\gamma (A\times \prod _{i=1}^\infty \mathbb {R}^d) = \mu (A)$ for all Borel $A\subset \mathbb {R}^d$. A key object of interest is the optimal cost of the problem (1.12) as a function of the marginal measure,

1.13 $$\begin{aligned} {F}^{OT}_\infty [\mu ] = \inf _{\gamma \in {\mathcal {P}}_{sym}^\infty (\mathbb {R}^d),\, \gamma \mapsto \mu } C_\infty [\gamma ]. \end{aligned}$$

Because of the appearance of the infinite particle configurations $(x_1,\ldots ,x_N,\ldots )$ and of an infinite-body cost, we call the problem (1.12) an *infinite-body (or infinite-marginal) optimal transport problem*.

The large-$N$ limit of the DFT functional $V_{ee}^{SCE}$ described in the Introduction corresponds to the case $d=3$ and the *Coulomb cost* $c(x,y)=\frac{1}{|x-y|}$. In this case, the functional (1.13) becomes

1.14 $$\begin{aligned} {F}^{OT}_\infty [\mu ]&:= \inf _{\gamma \in {\mathcal {P}}_{sym}^\infty (\mathbb {R}^3),\,\gamma \mapsto \mu } \lim _{N\rightarrow \infty }\frac{1}{{N\atopwithdelims ()2}}C_N[\gamma ],\nonumber \\ {C}_N[\gamma ]&= \int _{(\mathbb {R}^3)^\infty } \sum _{1\le i<j\le N} \frac{1}{|x_i-x_j|} d\gamma (x_1,\ldots ,x_N,\ldots ). \end{aligned}$$

Our first main result is the following. Here and below, $\hat{f}$ denotes the Fourier transform of the function $f\in L^1(\mathbb {R}^d)$, defined by $\hat{f}(k)=\int _{\mathbb {R}^d} e^{-ik\cdot x} f(x)\, dx$, and $C_b(\mathbb {R}^d)$ denotes the space of bounded continuous functions on $\mathbb {R}^d$. We recall that for any $\mu \in \mathcal{P}(\mathbb {R}^d)$, the infinite product measure $\mu ^{\otimes \infty }$ is defined as the unique probability measure on $(\Omega _\infty ^d, \mathcal{B}_\infty ^d)$ such that

$$\begin{aligned} \mu ^{\otimes \infty }(A_1\times \cdots \times A_n\times \mathbb {R}^d\times \mathbb {R}^d\times \cdots )=\mu (A_1)\times \cdots \times \mu (A_n) \end{aligned}$$

for any $n\ge 1$ and for any Borel sets $A_i\in \mathbb {R}^d, i=1,2,\ldots ,n$. (For more information on product spaces and product measures on infinite spaces see for example Chapter 2.2.4 in [64].)

#### **Theorem 1.2**

(Mean field theory as exact solution to infinite-body optimal transport)

- Let $c \, : \, \mathbb {R}^{2d}\rightarrow \mathbb {R}_+\cup \{\infty \}$ in (1.10) be of the form $c(x,y) =\ell (x-y)$, where $\ell (z)=\ell (-z)$ for all $z\in \mathbb {R}^d$ (i.e. $c$ is symmetric), and either
  (i) $$\ell \in L^1(\mathbb {R}^d)\cap C_b(\mathbb {R}^d)$$, $$\hat{\ell }\ge 0$$ or
  (ii) $d=3$, $\ell (z)=1/|z|$ (Coulomb cost).
  Let $\mu \in \mathcal{P}(\mathbb {R}^d)$ be a measure such that
  1.15 $$\begin{aligned} \int _{\mathbb {R}^{2d}} c(x,y) \, \mu (dx)\mu (dy)<\infty . \end{aligned}$$
  Then the infinite-dimensional product measure on $(\mathbb {R}^d)^\infty$
  1.16 $$\begin{aligned} \gamma _0=\mu ^{\otimes \infty }=\mu \otimes \mu \otimes \cdots \end{aligned}$$
  is a minimizer of the infinite-body optimal transport problem (1.12), and the optimal cost is the mean field functional, i.e.
  1.17 $$\begin{aligned} {F}^{OT}_{\infty }[\mu ]=\int _{\mathbb {R}^{2d}} c(x,y) \, \mu (dx)\mu (dy). \end{aligned}$$
- If in addition $\hat{\ell }(z)$ is strictly bigger than zero for all $z$, then the independent measure (1.16) is the unique minimizer of the problem (1.12).

Note that in case (ii), i.e. the Coulomb cost in dimension $d=3$, the strict positivity condition $\hat{\ell }>0$ holds, because $\hat{\ell }(k)=4\pi /|k|^2$. Moreover by simple estimates (see e.g. eq. (5.21) in the proof of Theorem 5.6 in [17]) the finiteness condition in (a) holds for all $\mu \in L^1(\mathbb {R}^3)\cap L^3(\mathbb {R}^3)$; the latter is the natural $L^p$ type space into which the domain of the Hohenberg-Kohn functional embeds. As a consequence, the above results are valid for all densities of physical interest in DFT. However the Coulomb cost it is neither continuous nor does it belong to $L^1$. The obvious task to weaken the regularity assumptions in (i) so as to naturally include the Coulomb cost does not seem to be straightforward and lies beyond the scope of this article.

Our result stands in surprising contrast to the recent results in [52, 53] by one of us. For a class of costs including the many-body quadratic cost $\sum _{i \ne j}|x_i-x_j|^2$ studied by Gangbo and Swiech [32], the optimizer of the infinite-body OT problem is demonstrated to be a Monge type solution; that is, any two of the variables are completely dependent, rather than completely independent as is the case for our class of costs. This dichotomy exposes a fascinating sensitivity to the cost function in infinite-body optimal transport problems. This difference is not present in two-marginal problems, where fairly weak conditions on the cost which include both the quadratic and the Coulomb cost suffice to ensure Monge type solutions. A milder version of the dichotomy does however arise in the multi-body context, where for certain costs the solution can concentrate on high dimensional submanifolds of the product space [13, 50]. It does not seem to be until one gets to the infinite marginal setting, however, that complete independence of the variables becomes optimal for certain costs. The difference between the costs in our paper and those in [52, 53] can be expressed succinctly as positivity of the Fourier transform of $\ell$. Note that the latter is equivalent to the fact that $c(x,y)=\ell (x-y)$ is a *positive kernel*, i.e. associated integral operator $K\varphi (x):=\int _{\mathbb {R}^d}c(x,y)\varphi (y)\, dy$ satisfies $\langle \varphi , K\varphi \rangle \ge 0$ for all $\varphi \in C_0^\infty (\mathbb {R}^d)$. See Example 2.13(ii) in Sect. 2.2 for a simple explicit example of a cost function which satisfies all the assumptions in Theorem 1.2 except positivity of the Fourier transform and for which the conclusion of the theorem fails.

The basic idea for the proof of Theorem 1.2 is to represent the competing infinite-dimensional probability measures in (1.13) via de Finetti’s theorem, and identify the functional $C_\infty$ introduced in (1.12), with the help of Fourier transform calculus and elementary probability theory, as a sum of the mean field functional and a certain variance term minimized by completely independent measures.

Our second main result clarifies the relationship between the infinite-body optimal transport problem (1.12) and the corresponding $N$-body optimal transportation problem:

1.18 $$\begin{aligned} \text{ Minimize } \tilde{C}_N[\gamma _N]&:= \int _{\mathbb {R}^{dN}} \sum _{1\le i<j\le N}c(x_i,x_j)\, d\gamma _N(x_1,x_2\ldots ,x_N) \text{ over } \gamma _N\in {\mathcal {P}}^N_{sym} (\mathbb {R}^d)\nonumber \\&\text{ satisfying } \gamma _N\mapsto \mu . \end{aligned}$$

Here and below ${\mathcal {P}}^N_{sym}(\mathbb {R}^d)$ denotes the set of Borel probability measures $\gamma _N$ on $\mathbb {R}^{Nd}$ which are symmetric, i.e. satisfy Eq. (1.3) (with $\mathbb {R}^3$ replaced by $\mathbb {R}^d$). The optimal cost per particle pair as a function of the marginal measure will be denoted by $F^{OT}_N[\mu ]$; that is to say, for arbitrary $\mu \in {\mathcal {P}}(\mathbb {R}^{d})$ we set

1.19 $$\begin{aligned} {F}^{OT}_N[\mu ] := \frac{1}{{N\atopwithdelims ()2}} \inf _{\gamma _N\in {\mathcal {P}}_{sym}^N(\mathbb {R}^{d}),\gamma _N\,\mapsto \mu }{\tilde{C}}_N[\gamma _N]. \end{aligned}$$

We show:

#### **Theorem 1.3**

(N-body cost approaches infinite-body cost) Assume that $\mu \in {\mathcal {P}}(\mathbb {R}^d)$ is a probability measure such that there exists a measure $\gamma _0\in {\mathcal {P}}_{sym}^\infty (\mathbb {R}^{d})$ with $\gamma _0\mapsto \mu$ and $\int _{(\mathbb {R}^d)^\infty } c(x_1,x_2)d\gamma _0(x_1,x_2,\ldots )<\infty$. Let the cost function $c \, : \, \mathbb {R}^{2d}\rightarrow [0,\infty )\cup \{+\infty \}$ in (1.18) and (1.11) be Borel-measurable, symmetric, and either (i) bounded, or (ii) lower semi-continuous as a map with values into $[0,\infty )\cup \{+\infty \}$ endowed with its natural topology; ie, $c(x_j) \rightarrow \infty$ whenever $x_j \rightarrow x$ and $c(x) = \infty$. Then we have

1.20 $$\begin{aligned} F^{OT}_\infty [\mu ]=\lim _{N\rightarrow \infty } {F}^{OT}_N[\mu ]. \end{aligned}$$

Note that here not just costs leading to independence as in Theorem 1.2 but also costs leading to strong correlations as considered in [52, 53] are included.

The proof of Theorem 1.3 is based on a construction from advanced probability theory [22] which does not appear to be easily accessible to non-probabilists, and which contains the important insight that any $N$-body measure $\gamma _N\in {\mathcal {P}}^N_{sym}(\mathbb {R}^{d})$ can be approximated by the $N$-body marginal $\tilde{\gamma }_N$ of an infinite probability measure $\gamma \in {\mathcal {P}}_{sym}^\infty (\mathbb {R}^d)$ ($\tilde{\gamma }_N$ is infinitely representable in the terminology developed below). This allows us to approximate the $N$-body OT problem (1.18) as arising in density functional theory by the corresponding infinite-body OT problem (1.12). Interestingly, the focus of probabilists was precisely the other way around: the object of primary interest were the infinite probablity measures in the space ${\mathcal {P}}_{sym}^\infty$, or in fact the underlying infinite sequences of random variables. The latter serve as useful alternatives to iid (identically and independently distributed) sequences which allow to model repeated sampling experiments containing correlations; approximation by finite sequences of random variables was then of interest for purposes of numerical sampling.

Finally let us describe what our results imply for the SCE functional (1.1), (1.2) arising in density functional theory. Roughly, they allow to analyze a natural *inhomogeneous high-density limit* in which the inhomogeneity is not a small perturbation, but stays proportional to the overall density. More precisely, one fixes an arbitrary density $\mu$ of integral $1$, considers the $N$-body system with proportional inhomogeneity, i.e. with one-body density given by $\rho =N\mu$, and studies the asymptotics of the SCE energy as $N$ gets large. Note that the SCE energy corresponds, up to normalization factors, to the optimal cost functional (1.19) with Coulomb cost $c(x,y)=1/|x-y|$ in dimension $d=3$:

1.21 $$\begin{aligned} V_{ee}^{SCE}[\rho ] = {N \atopwithdelims ()2} F_N^{OT}\left[ \frac{\rho }{N}\right] . \end{aligned}$$

Combining Theorems 1.2 and 1.3 immediately yields:

#### **Corollary 1.4**

(Inhomogeneous high-density limit of the SCE functional) Let $\mu \, : \, \mathbb {R}^3\rightarrow \mathbb {R}$ be any nonnegative function with $\int _{\mathbb {R}^3}\mu =1$ which belongs to $L^1(\mathbb {R}^3)\cap L^3(\mathbb {R}^3)$. Let $\rho ^{(N)}=N\mu$. Then as $N$ gets large, the SCE energy of $\rho ^{(N)}$ is asymptotic to the mean field energy, that is to say

$$\begin{aligned} \lim _{N\rightarrow \infty } \frac{V_{ee}^{SCE}[\rho ^{(N)}]}{J[\rho ^{(N)}]} = 1, \end{aligned}$$

where $J$ is the functional (1.9).

We remark that both numerator and denominator are of order $N^2$ as $N\rightarrow \infty$, i.e. they are proportional to the number of particle pairs in the system. A very interesting question raised by our work is to determine asymptotic corrections to the mean field energy in Eq. (1.20). For non-singular costs, we expect the next-order correction to occur at the thermodynamic order $O(N)$. Unfortunately, understanding these corrections lies beyond the scope of the methods developed here.

#### *Remark 1.5*

A very interesting alternative proof of the preceding corollary for the Coulombic cost function was pointed out to us by Paola Gori-Giorgi. This proof, and hence also the above corollary, is implicit in recent work in the physics literature [57]. The key ingredient is a nontrivial Coulombic inequality, the Lieb–Oxford bound [48], The argument is as follows: the Lieb–Oxford bound, in our notation, states that

$$\begin{aligned} V_{ee}^{SCE}[\rho ^{(N)}]-J[\rho ^{(N)}] \ge -C \int _{\mathbb {R}^3} (\rho ^{(N)})^{4/3}, \end{aligned}$$

for some constant $C$ independent of $N$. (Strictly speaking, the bound was only formulated and derived in [48] for $N$-point densities which arise from some wavefunction, but the proof generalizes easily to probability measures.) Noting that the left hand side is non positive (by using the independent $N$-point density as trial function in the variational principle for $V_{ee}^{SCE}$), and that $V_{ee}^{SCE}[\rho ^{(N)}]$ and $J[\rho ^{(N)}]$ scale like $N^2$ while $\int _{\mathbb {R}^3} (\rho ^{(N)})^{4/3} = N^{4/3}\int _{\mathbb {R}^3} \mu ^{4/3}$ scales as $N^{4/3}$, we divide by $J[\rho ^{(N)}]$ and let $N$ tend to $\infty$ to obtain the desired result.

The arguments developed in the present paper apply to a larger class of interaction energies (see Definition 1.1, Theorem 1.2), and—perhaps more importantly—are based on a general and transparent probabilistic inequality (namely the comparison estimate in Proposition 3.2 below between infinitely representable and finitely representable measures which goes back to Diaconis and Freedman). By contrast the Lieb–Oxford inequality was derived using highly nontrivial ad hoc estimates and currently lacks a probabilistic interpretation and analogues for non-Coulombic problems. But—unlike the Lieb–Oxford inequality—our probabilistic arguments fail to give a quantitative error bound for the associated optimal cost functionals for singular costs like the Coulomb cost, yielding such bounds only in the case of bounded costs (see Eq. (3.6)).

### Plan of paper

The rest of the paper is organized as follows. In Sect. 2 we recall the notion of $N$-representability of pair measures, which was developed in the present OT context in our recent paper [27] and is equivalent to the concept of $N$-extendability of pairs of random variables in probability theory, and prove Theorem 1.2. Section 3 is devoted to the proof of Theorem 1.3.

## Solution to the infinite-body OT problem

The proof of Theorem 1.2 will require two key Lemmas. The first one (Lemma 2.4) reduces the infinite-body OT problem (1.12) to a $2$-body OT problem with an infinite representability constraint. The second (Lemma 2.8) gives an explicit description of the measures satisfying this infinite representability constraint (de Finetti’s Theorem, stated in Proposition 2.7 below).

In Sect. 2.1 we recall the notion of $N$-representability of a pair density, generalize it to infinitely many particles, prove Lemmas 2.4 and 2.8, and also establish existence of at least one solution to (1.12) (Proposition 2.9). In Sect. 2.2 we establish Theorem 1.2, via Fourier transform calculus applied to the de Finetti representation of infinitely representable measures.

### Reduction to a $2$-body OT problem with infinite representability constraint

We now reformulate the infinite-body mass transportation problem (1.12) as a standard (two-body) mass transportation problem subject to an infinite representability constraint. This reformulation is possible due to the fact that the cost in (1.10) is a sum of symmetric pair terms. We begin by recalling the definition of $N$-representability, introduced in the present context in our recent paper [27] (see Definition III.1).

#### **Definition 2.1**

(N-representability) Let $N\ge 2$. A symmetric probability measure $\mu _2\in {\mathcal {P}}_{sym}^2(\mathbb {R}^{d})$ is said to be $N$ *-representable* if there exists a symmetric probability measure $\gamma _N\in {\mathcal {P}}_{sym}^N(\mathbb {R}^{d})$ such that for all Borel sets $A_i,A_j\subseteq \mathbb {R}^d$ and all $1\le i<j\le N$, we have

2.1 $$\begin{aligned} \gamma _N(\mathbb {R}^{d(i-1)}\times A_i \times \mathbb {R}^{d(j-(i-1))}\times A_j\times \mathbb {R}^{d(N-(j-1))}) = \mu _2(A_i\times A_j). \end{aligned}$$

$N$-representability is a highly nontrivial restriction. The following basic example is taken from [27].

#### *Example*

Let $A$, $B\in \mathbb {R}^d$, $A\ne B$. The totally anticorrelated probability measure $\mu _2 = \frac{1}{2}(\delta _A\otimes \delta _B + \delta _B\otimes \delta _A)$ is not 3-representable. (Here $\delta _A$ denotes the Dirac measure centred at $A$.)

Intuitively, this is because we can not allocate 3 particles to 2 sites without doubly occupying one of the sites. Mathematically, to prove this suppose that $\gamma$ was any probability measure on $(\mathbb {R}^d)^3$ with two-body marginal $\mu _2$. Then $\gamma$ must have one-body marginal supported on $\{A,B\}$, and hence must be a convex combination of the measures $\delta _X\otimes \delta _Y\otimes \delta _Z$ with $X,Y,Z\in \{A,B\}$. But the two-point marginal of each of the latter measures contains a positive multiple of either $\delta _A\otimes \delta _A$ or $\delta _B\otimes \delta _B$, whence the two-pont marginal of $\gamma$ cannot equal $\mu _2$. For further discussion and more general examples we refer to [27].

Two quantum analogues of $N$-representability are widely studied in the physics and quantum chemistry literature. The first one, (wavefunction) representability of a pair density, is closely related to the notion above and asks whether a symmetric nonnegative function $p_2\, : \, \mathbb {R}^{2d}\rightarrow \mathbb {R}$ of unit integral satisfies

$$\begin{aligned} p_2(x_1,x_2)=\sum _{s_1,\ldots ,s_N\in \mathbb {Z}_2} \int _{\mathbb {R}^{d(N-2)}}|\Psi (x_1,s_1,x_2,s_2,\ldots ,x_N,s_N)|^2 \end{aligned}$$

for some square-integrable antisymmetric normalized $N$-electron wavefunction $\Psi \, : \, (\mathbb {R}^d\times \mathbb {Z}_2)^N\rightarrow \mathbb {C}$. Wavefunction representability trivially implies representability in the sense of the definition above. Conversely, many known necessary conditions on representability by an $N$-electron wavefunction, such as the Davidson [19] and generalized Davidson [1] constraints, continue to hold for pair densities which are $N$-representable in the sense of Definition 2.1, as their derivation in fact only uses representability by a symmetric probability measure.

In the second quantum analogue, one asks whether a function $\Gamma \, : \, (\mathbb {R}^d\times \mathbb {Z}_q)^4 \rightarrow \mathbb {C}$ is of the form

2.2 $$\begin{aligned} \Gamma (z_1,z_2; z_1',z_2') = \int _{\mathbb {R}^{(N-2)d}} \Psi (z_1,z_2,z_3,\ldots ,z_N)\overline{\Psi (z_1',z_2',z_3,\ldots ,z_N)} \, dz_3,\ldots , dz_N\nonumber \\ \end{aligned}$$

for some antisymmetric function $\Psi \in L^2((\mathbb {R}^d\times \mathbb {Z}_q)^N)$ of unit norm, with the case of electrons corresponding to $d=3$, $q=2$. Mathematically, $\Gamma$ should be viewed as a unit-trace operator $\hat{\Gamma }$ on the two-body Hilbert space $L^2((\mathbb {R}^d\times \mathbb {Z}_q)^2)$, acting as

$$\begin{aligned} \varphi \mapsto (\hat{\Gamma }\varphi )(z_1,z_2) = \int _{(\mathbb {R}^d\times \mathbb {Z}_q)^2} \Gamma (z_1,z_2;z_1',z_2') \varphi (z_1',z_2')\, dz_1' dz_2'. \end{aligned}$$

Eq. (2.2) means that $\hat{\Gamma }$ can be represented as a partial trace of the unit-trace operator $|\Psi \rangle \langle \Psi |$ on the $N$-body Hilbert space $L^2((\mathbb {R}^d\times \mathbb {Z}_q)^N)$. For an overview of results on the quantum representability problem we refer to [14].

The notion of $N$-representability in Definition 2.1 is well known in the probability theory literature, under the names *N-extendability* or *finite exchangeability*, and is usually stated and analyzed in the language of sequences $X_1,\ldots ,X_N$ of $N$ random variables. The formulation in Definition 2.1 is mathematically equivalent and corresponds to considering instead the law of the random vector $(X_1,\ldots ,X_N)$. Numerous attempts have been made to characterize $N$-extendability for $N\ge 3$ for various types of marginals (see, for example, [2] for an an in-depth overview of $N$-extendability results in probability), but a direct characterization remains elusive.

Let us now generalize Definition 2.1 to infinite particle systems.

#### **Definition 2.2**

(Infinite representability) Analogously to the $N$-representability case, a symmetric probability measure $\mu _2\in {\mathcal {P}}_{sym}^2(\mathbb {R}^{d})$ is said to be *infinitely representable* if there exists a symmetric probability measure $\gamma _\infty \in {\mathcal {P}}_{sym}^\infty (\mathbb {R}^d)$ such that for all Borel sets $A_i,A_j\subseteq \mathbb {R}^d$ and all $1\le i<j\le N$, we have

2.3 $$\begin{aligned} \gamma _\infty (\mathbb {R}^{d(i-1)}\times A_i \times \mathbb {R}^{d(j-(i-1))}\times A_j\times \mathbb {R}^d\times \cdots \times \mathbb {R}^d\times \cdots ) = \mu _2(A_i\times A_j). \end{aligned}$$

Note that a symmetric probability measure $\gamma _\infty \in {\mathcal {P}}_{sym}^\infty (\mathbb {R}^d)$ is called an *exchangeable* measure in the probabilistic literature. It is easy to see (see, for example, [2] or Lemma III.2 in [27]) that

#### **Lemma 2.3**

Let $N\ge M \ge 2$. If $\mu _2\in {\mathcal {P}}_{sym}^2(\mathbb {R}^{d})$ is $N$-representable, then it is also $M$-representable.

That is to say, $N$-representability becomes a more and more stringent condition as $N$ increases. Note that the case $N=\infty$ will be studied later in Lemma 3.3.

We will next reformulate the minimization problem (1.12) in terms of infinite representability. The result is a straightforward extension to infinite particle systems of Theorem III.3 in [27] for the $N$-body problem.

#### **Lemma 2.4**

For any $\mu \in {\mathcal {P}}(\mathbb {R}^d)$ we have

2.4 $$\begin{aligned} {F}^{OT}_{\infty }[\mu ]&= \inf \Bigg \{\int _{\mathbb {R}^{2d}} c(x,y) \, d\mu _2(x,y) \; \Big | \; \nonumber \\&\mu _2\in {\mathcal {P}}_{sym}^2(\mathbb {R}^{d}), \, \mu _2\mapsto \mu , \, \mu _2 \text{ is } \text{ infinitely } \text{ representable } \Bigg \}. \end{aligned}$$

#### *Proof*

This is clear from the observation that ${C}_{\infty }[\gamma ] = \int _{\Omega ^2}cd\mu _2$ for any $\gamma \in {\mathcal {P}}_{sym}^\infty (\mathbb {R}^d)$ with $\gamma \rightarrow \mu _2$ and definition of infinite representability. $\square$

To prove our next result, we will use the de Finetti–Hewitt–Savage Theorem for infinitely representable measures as stated and proved in [22]. See [22] Theorems 14 and 20 (for exchangeable measures in ${\mathcal {P}}_{sym}^\infty (\mathcal{S})$, where $\mathcal{S}$ is a compact Hausdorff space) and the third paragraph on page 751 (for the more general case of standard spaces $\mathcal{S}$ as defined in Definition 2.5 below). Note that de Finetti’s Theorem can be found in the literature—under various assumptions on $\mathcal{S}$—in the different but equivalent language of exchangeable sequences of random variables on $\mathcal{S}$, starting with the seminal paper of [20] (for exchangeable Bernoulli random variables). The statement of the theorem requires some more notation and definitions.

#### **Definition 2.5**

Two probability spaces $(\Upsilon _1, \mathcal{G}_1, {\mathbb {P}}_1)$ and $(\Upsilon _2, \mathcal{G}_2, {\mathbb {P}}_2)$ are *isomorphic* if there exists a bijective map $T \, : \Upsilon _1\rightarrow \Upsilon _2$ such that $T$ and $T^{-1}$ map measurable sets to measurable sets and are measure preserving.

Two probability spaces $(\Upsilon _1, \mathcal{G}_1, {\mathbb {P}}_1)$ and $(\Upsilon _2, \mathcal{G}_2, {\mathbb {P}}_2)$ are *isomorphic mod* $0$ if there exist null sets $A_1\subset \Upsilon _1, A_2\subset \Upsilon _2$ such that the probability spaces $\Upsilon _1{\setminus }A_1, \Upsilon _2{\setminus }A_2$ (endowed with the natural sigma-fields and probability measures) are isomorphic.

A probability space is called a *standard space* if it is isomorphic mod $0$ to an interval with Lebesgue measure, a finite or countable sum of atoms (i.e. measures whose support consists of a single point), or a disjoint union of both.

For further discussion of the notion of standard space and examples see [40].

Let $(S,\mathcal{B}(\mathcal{S}))$ be an abstract measurable space.

#### *Remark 2.6*

The main point for our purposes is that $(\mathcal{S}, \mathcal{B}(\mathcal{S}), \mu )$ is a standard space provided $S$ is Polish (i.e., a complete separable metric space), $\mathcal{B}(\mathcal{S})$ is the Borel $\sigma$-field, and $\mu$ is any Borel probability measure on $(\mathcal{S}, \mathcal{B}(\mathcal{S}))$. In particular, $\mathbb {R}^d$ and $(\mathbb {R}^d)^\infty$ endowed with any Borel probability measure are standard spaces. This follows e.g. by combining Theorem 2.4.1 in [40], which establishes that any Polish space endowed with a regular probability measure is standard, and the general measure-theoretic fact (see e.g. [5]) that any Borel probability measure on a Polish space is regular.

We endow $\mathcal{P}(\mathcal{S})$—the set of all probability measures on $(\mathcal{S}, \mathcal{B}(S))$—with the smallest $\sigma$-algebra $\mathcal{{B}}^*(\mathcal{S})$ which makes the functions $\mathbb {P}\rightarrow \mathbb {P}(A), \mathbb {P}\in \mathcal{P}(\mathcal{S}),$ measurable for all $A\in \mathcal{B}(\mathcal{S})$. We note here that in the weak star topology of $\mathcal{P}(\mathcal{S})$ (in which the convergence is called weak convergence of measures), the map $\mathbb {P}\rightarrow \mathbb {P}(A), A\in \mathcal{B}(\mathcal{S}),$ is continuous, and therefore $\mathcal{{B}}^*(\mathcal{S})$ is by definition the Baire $\sigma$-field in $\mathcal{P}(\mathcal{S})$. If $\mathcal{S}$ is a metric space, the Baire $\sigma$-field $\mathcal{{B}}^*(\mathcal{S})$ coincides with the Borel $\sigma$-field on $\mathcal{P}(\mathcal{S})$. (For more on Baire and Borel $\sigma$-fields, see for example Chapter 6 in [6] or [22], and for more on the weak star topology of $\mathcal{P}(\mathcal{S})$ see Chapter 8 in [6].)

We are now ready to state de Finetti’s Theorem. Translated into the present language, de Finetti’s Theorem says the following:

#### **Proposition 2.7**

(De Finetti–Hewitt–Savage Theorem) Let $\mathcal{S}$ be $\mathbb {R}^d$, or more generally any space with the property that $(S, \mathcal{B}(\mathcal{S}), \mu )$ is a standard space for all Borel probability measures $\mu$, where $\mathcal{B}(\mathcal{S})$ is the Borel $\sigma$-field. Let $\mathcal{P}(\mathcal{S})$ be the set of probability measures on $(S, \mathcal {B}(\mathcal{S}))$, and let $\mathcal{{B}}^*(\mathcal{S})$ be the Borel $\sigma$-field in $\mathcal{P}(\mathcal{S})$. Let $\gamma _\infty$ be a symmetric Borel measure on the Borel $\sigma$-field $\mathcal{{B}}^\infty (\mathcal{S})$ of the cartesian product $\mathcal{S}^\infty$ (recall Definition 1.1 above). Then there exists a unique Borel probability measure $\nu$ on $\mathcal{{B}}^*(\mathcal{S})$ such that

2.5 $$\begin{aligned} \gamma _\infty =\int _{ \mathcal{P}(\mathcal{S})} Q^{\otimes \infty }d\nu (Q). \end{aligned}$$

In words: one can view an (infinite-dimensional) exchangeable probability measure $\gamma _\infty$ on $S^{\otimes \infty }$ as an integral of infinite product probability measures on $S^{\otimes \infty }$ against a probability measure defined on the space of all probability measures on $S$. An equivalent statement of De Finetti’s theorem is that the *extremal points* of the convex set of exchangeable probability measures on an infinite product space $S^{\otimes \infty }$ are the infinite-dimensional product measures $Q^{\otimes \infty }$ on $S^{\otimes \infty }$. De Finetti’s theorem asserts, moreover, that this convex set is a *simplex*, i.e. any of its points $\gamma _\infty$ is the barycentre of a unique probability measure $\nu$ concentrated on the extremal points $Q^{\otimes \infty }$.

Next we reformulate the optimal cost functional (1.13) with the help of de Finetti’s theorem. We will use that formula (2.5) means in particular that

$$\begin{aligned} \gamma _\infty (A_1\times \cdots \times A_n\times \mathbb {R}^d\times \mathbb {R}^d\times \cdots )=\int _{ \mathcal{P}(\mathcal{S})} Q(A_1)\times \cdots \times Q(A_n)d\nu (Q) \end{aligned}$$

for all $n\ge 1$ and for all Borel sets $A_i\in \mathcal{B}(\mathcal{S})$, $i=1,2,\ldots , n$.

#### **Theorem 2.8**

For any $\mu \in {\mathcal {P}}(\mathbb {R}^d)$, the functional $F_\infty ^{OT}[\mu ]$ introduced in (1.13) satisfies

2.6 $$\begin{aligned} {F}^{OT}_{\infty }[\mu ]&= \inf \Bigg \{\int _{\mathbb {R}^{2d}} c(x,y) \, d\mu _2(x,y) \; \Big | \; \mu _2=\int _{{\mathcal {P}}(\mathbb {R}^d)}Q\otimes Q \, d\nu (Q)\nonumber \\&~ \text{ and } \mu = \int _{{\mathcal {P}}(\mathbb {R}^d)}Q \, d\nu (Q) ~ \text{ for } \text{ some }~{\nu \in \mathcal P}(\mathcal{P }(\mathbb {R}^d))\Bigg \}. \end{aligned}$$

Moreover $\gamma =\int _{{\mathcal {P}}(\mathbb {R}^d)}Q^{\otimes \infty } d\nu (Q)$ is a minimizer of the problem (1.12) if and only if $\mu _2=\int _{{\mathcal {P}}(\mathbb {R}^d)} Q\otimes Q \, d\nu (Q)$ is a minimizer of the problem in (2.6).

#### *Proof*

Note that the one and two body marginals of $\gamma _{\infty }$ in (2.5) are given by $\mu = \int _{{\mathcal {P}}(\mathbb {R}^d)} Q \, d\nu (Q)$ and $\mu _2 = \int _{{\mathcal {P}}(\mathbb {R}^d)}Q\otimes Q \, d\nu (Q)$, respectively. Then, by de Finetti’s Theorem, $\mu _2$ is infinitely representable if and only if $\mu _2 = \int _{{\mathcal {P}}(\mathbb {R}^d)}Q\otimes Q \, d\nu (Q)$ for some $\nu \in \mathcal{P}(\mathcal{P}(\mathbb {R}^d))$. The result follows from Lemma 2.4. $\square$

We end this subsection with a general result of existence of at least one solution to (1.12) and to (2.4). This result will be used in the proof of Theorem 1.3.

#### **Theorem 2.9**

For all $N\in \mathbb {N}, N\ge 2$, let $c_N\, : \, (\mathbb {R}^d)^N \rightarrow {\mathbb {R}}_+\cup \{\infty \}$ be defined as in (1.10), with $c$ Borel-measurable, symmetric, and lower semi-continuous. Then there exists at least one solution $\gamma ^{opt}$ to (1.12) and at least one solution $\mu _2^{opt}$ to the minimization problem in (2.4).

#### *Proof*

To prove the existence of a solution $\gamma \in {\mathcal {P}}_{sym}^\infty (\mathbb {R}^d),\gamma \mapsto \mu$, to (1.12), we will adapt to our infinite-body optimal transportation problem the standard proof of existence of solutions to two-body OT problems as given e.g. in [65], Theorem 4.1. Since there are some subtle differences to the proof in [65], we will outline below the basic steps.

The proof relies on basic variational arguments involving the topology of weak convergence (imposed by bounded continuous test functions). There are two key properties on which the proof relies:

- Lower semicontinuity of the cost functional $\gamma \mapsto C_\infty [\gamma ]$ on ${\mathcal {P}}_{sym}^\infty (\mathbb {R}^d)$ with respect to weak convergence. This follows by a standard argument after rewriting $C_\infty [\gamma ] = \int _{\mathbb {R}^{2d}}c(x_1,x_2)d\mu _2(x_1,x_2)$ and by noting that the class of infinite-dimensional symmetric probability measures in ${\mathcal {P}}_{sym}^\infty (\mathbb {R}^d)$ is closed under weak convergence in the sense that if $\{P_k\in {\mathcal {P}}_{sym}^\infty (\mathbb {R}^d)\}_{k\ge 1}$ converges weakly to a probability measure $\mathbb {P}\in {{\mathcal {P}}}((\mathbb {R}^d)^\infty )$, then $\mathbb {P}\in {\mathcal {P}}_{sym}^\infty (\mathbb {R}^d)$ (for a proof of this statement, see e.g. page 54 in [2]).
- Tightness in ${\mathcal {P}}_{sym}^\infty (\mathbb {R}^d)$ of the set of all $\gamma \in {\mathcal {P}}_{sym}^\infty (\mathbb {R}^{d})$ such that $\gamma \mapsto \mu$ for some fixed $\mu \in {\mathcal {P}}(\mathbb {R}^{d})$. This is proved similarly to Lemma 4.3 from [65]. More precisely, let $\gamma \in {\mathcal {P}}_{sym}^\infty (\mathbb {R}^{d})$ such that $\gamma \mapsto \mu$ and $\mu \in {\mathcal {P}}(\mathbb {R}^{d})$. Since $\mathbb {R}^d$ is a Polish space, $\mu$ is tight in ${\mathcal {P}}(\mathbb {R}^d)$. Then for any $\epsilon >0$ and for any $i\in \mathbb {N}, i\ge 1$, there exists a compact set $K^i_\epsilon \subset \mathbb {R}^d$, independent of the choice of $\gamma$, such that $\mu (\mathbb {R}^d\setminus K^i_\epsilon ) \le \frac{\epsilon }{2^i}$. Take $K_\epsilon :=\prod _{i\ge 1}K^i_\epsilon$, which is compact by Tychonoff’s theorem. Then we have
  $$\begin{aligned} \gamma (K_\epsilon ^c)\le \gamma (\cup _{i\ge 1}(\underbrace{\mathbb {R}^d\times \cdots \times \mathbb {R}^d}_\mathrm{i-1 times }\times (K_\epsilon ^i)^c\times \mathbb {R}^d\times \cdots ))\le \sum _{i\ge 1}\mu ((K_\epsilon ^i)^c)\le \sum _{i\ge 1}\frac{\epsilon }{2^i}=\epsilon . \end{aligned}$$
  Tightness now follows since this bound is independent of $\gamma$.

Given (a) and (b), the existence of a solution ${\gamma }^{opt}$ to (1.12) follows analogously to the proof of Theorem 4.1 from [65]: take a minimizing sequence $(\gamma ^\alpha )_\alpha$, extract a weakly convergent subsequence via (b) and Prokhorov’s theorem, and pass to the limit via (a).

One now trivially also obtains a solution to the variational problem in (2.4); namely, the two-point marginal $\mu _2^{opt}$ of $\gamma ^{opt}$ is a solution. $\square$

### Proof of Theorem 1.2

In this subsection, we determine explicitly the optimal transport functional ${F}^{OT}_\infty$ introduced in Eq. (1.12), for a large class of cost functions. As an offshot, we obtain an interesting probabilistic interpretation of the infinite-body optimal transport functional $C_\infty$ introduced in (1.12).

#### *Proof of Theorem 1.2*

We will show explicitly that

2.7 $$\begin{aligned} \int _{\mathbb {R}^{2d}} c(x,y) \, d\mu _2(x,y) \ge \int _{\mathbb {R}^{2d}} c(x,y) d\mu (x)d\mu (y) \end{aligned}$$

for any $\mu _2 =\int Q\otimes Qd\nu (Q)$ with $\nu \in \mathcal{P}(\mathcal{P}(\mathbb {R}^d))$, and, if $\hat{l}>0$ everywhere, equality can only hold when $\mu _2 =\mu \otimes \mu$ is product measure. The result then follows easily from Theorem 2.8.

The central idea is to re-write both terms in (2.7) using Fourier calculus and elementary probability theory. For any $Q\in {\mathcal {P}}(\mathbb {R}^d)$ such that $\int _{\mathbb {R}^{2d}}\ell (x-y)dQ(x)dQ(y)<\infty$, let $\ell *Q$ and $\hat{Q}$ denote, respectively, the convolution of $\ell$ and $Q$ and the Fourier transform of $Q$, i.e.

$$\begin{aligned} (\ell *Q)(x ):=\int _{\mathbb {R}^d}\ell (x-y)dQ(y),~~\hat{Q}(z)=\int _{\mathbb {R}^d}e^{-i z\cdot x} dQ(x). \end{aligned}$$

The first function may take the value $+\infty$, whereas the second is a bounded continuous function on $\mathbb {R}^d$. In order not to obscure the main argument, we first calculate the integral in (2.6) *formally*, using the rules of Fourier transform calculus even though $\ell$ and $Q$ are not smooth rapidly decaying functions. The calculation will be justified rigorously in Lemma 2.10 below. Using, in order of appearance, Fubini’s theorem, the definition of the convolution, Plancherel’s formula, the Fourier calculus rule $\widehat{f*g}=\hat{f} \, \hat{g}$, and again Fubini’s theorem gives, abbreviating $c_d:=(2\pi )^{-d}$,

2.8 $$\begin{aligned} \int _{\mathbb {R}^{2d}} c(x,y) d\mu _2(x,y)&= \int _{\mathbb {R}^{2d}} \ell (x-y)\int _{{\mathcal {P}}(\mathbb {R}^d)}dQ(x)dQ(y)d\nu (Q)\nonumber \\&= \int _{{\mathcal {P}}(\mathbb {R}^d)}\int _{\mathbb {R}^{2d}}\ell (x-y)\, dQ(x)\, dQ(y)\, d\nu (Q)\nonumber \\&= \int _{{\mathcal {P}}(\mathbb {R}^d)}\int _{\mathbb {R}^{d}}(\ell *Q)(x)\, dQ(x)\, d\nu (Q)\nonumber \\&= c_d\int _{{\mathcal {P}}(\mathbb {R}^d)}\int _{\mathbb {R}^{d}}(\widehat{\ell *Q})(z)\bar{\hat{Q}}(z)\, dz \, d\nu (Q)\nonumber \\&= c_d\int _{{\mathcal {P}}(\mathbb {R}^d)}\int _{\mathbb {R}^{d}}\hat{\ell }(z)|\hat{Q}(z)|^2 dz\, d\nu (Q)\nonumber \\&= c_d\int _{\mathbb {R}^{d}} \hat{\ell }(z) \int _{{\mathcal {P}}(\mathbb {R}^d)}|\hat{Q}(z)|^2d\nu (Q)dz. \end{aligned}$$

By a similar reasoning, we have

2.9 $$\begin{aligned} \int _{\mathbb {R}^{2d}} c(x,y) d\mu (x)d\mu (y)&= \int _{\mathbb {R}^{2d}} \ell (x-y)\int _{{\mathcal {P}}(\mathbb {R}^d)\times {\mathcal {P}}(\mathbb {R}^d)}dQ(x)\, d\nu (Q)\, d\tilde{Q}(y) d\nu (\tilde{Q})\nonumber \\&= \int _{{\mathcal {P}}(\mathbb {R}^d)\times {\mathcal {P}}(\mathbb {R}^d)}\int _{\mathbb {R}^{2d}}\ell (x-y)dQ(x)d\tilde{Q}(y)d\nu (Q)d\nu (\tilde{Q})\nonumber \\&= \int _{{\mathcal {P}}(\mathbb {R}^d)\times {\mathcal {P}}(\mathbb {R}^d)} \int _{\mathbb {R}^{d}}(\ell *\tilde{Q})(x)dQ(x)d\nu (Q)d\nu (\tilde{Q})\nonumber \\&= c_d\int _{{\mathcal {P}}(\mathbb {R}^d)\times {\mathcal {P}}(\mathbb {R}^d)}\int _{\mathbb {R}^{d}}(\widehat{\ell *\tilde{Q}})(z)\bar{\hat{Q}}(z)dz\, d\nu (Q)\, d\nu (\tilde{Q})\nonumber \\&= c_d\int _{{\mathcal {P}}(\mathbb {R}^d)\times {\mathcal {P}}(\mathbb {R}^d)} \int _{\mathbb {R}^{d}}\hat{\ell }(z)\hat{\tilde{Q}}(z)\bar{\hat{Q}}(z)\, dz\, d\nu (Q)\, d\nu (\tilde{Q})\nonumber \\&= c_d\int _{\mathbb {R}^{d}}\hat{\ell }(z)\Big |\int _{P(\mathbb {R}^d)}\hat{Q}(z)\, d\nu (Q)\Big |^2dz. \end{aligned}$$

Finally, decomposing the expressions on the right hand side of (2.8) and (2.9) into their real and imaginary part gives the formal identity

2.10 $$\begin{aligned}&\int _{\mathbb {R}^{2d}} \ell (x-y) d\mu _2(x,y) - \int _{\mathbb {R}^{2d}} \ell (x-y) d\mu (x)d\mu (y) \nonumber \\&\quad = c_d\int _{\mathbb {R}^d}\hat{\ell }(z)\left[ \int _{P(\mathbb {R}^d)} (Re(\hat{Q}(z)))^2d\nu (Q)-{\left( \int _{P(\mathbb {R}^d)}Re(\hat{Q}(z))d\nu (Q)\right) ^2}\right] dz \nonumber \\&\qquad + c_d\int _{\mathbb {R}^d}\hat{\ell }(z)\left[ \int _{P(\mathbb {R}^d)} (Im(\hat{Q}(z)))^2d\nu (Q)-{\left( \int _{P(\mathbb {R}^d)}Im(\hat{Q}(z))d\nu (Q)\right) ^2}\right] dz \nonumber \\&\quad = c_d\int _{\mathbb {R}^d}\hat{\ell }(z)\left( \mathrm{var}_{\nu (dQ)}Re(\hat{Q}(z)) + \mathrm{var}_{\nu (dQ)}Im(\hat{Q}(z))\right) dz. \end{aligned}$$

Here $Re(\hat{Q}(z))$ and $Im(\hat{Q}(z))$ denote the real and the imaginary parts of $\hat{Q}(z)$, and $\mathrm{var}_{\nu (dQ)}Re(\hat{Q}(z))$ and $\mathrm{var}_{\nu (dQ)}Im(\hat{Q}(z))$ are the variances of the random variables $Re(\hat{Q}(z))$ and $Im(\hat{Q}(z))$ with respect to the probability measure $\nu (dQ)$.

The only steps in the derivation of (2.8), (2.9), (2.10) which were nonrigorous due to lack of regularity of $\ell$ and $Q$ were the use of Plancherel’s formula and of the Fourier calculus rule $\widehat{\ell *Q}=\hat{\ell }\hat{Q}$. Conventional assumptions would be $\ell *Q$ and $Q\in L^2(\mathbb {R}^d)$ for the former, and $\ell$ and $Q\in L^1(\mathbb {R}^d)$ for the latter. As none of these four assumptions are actually met here, we will need the following generalization of these facts. Though this will surely not be surprising to experts in the interest of completeness and for lack of a suitable reference, we include a proof in the “Appendix”.

#### **Lemma 2.10**

If either $\ell \in C_b(\mathbb {R}^d)\cap L^1(\mathbb {R}^d)$, $\hat{\ell }\ge 0$, or $\ell$ is the Coulomb cost $\ell (z)=1/|z|$ in dimension $d=3$, and $\int \ell (x-y)\, dQ(x)\, dQ(y)<\infty$, $\int \ell (x-y)\, d\tilde{Q}(x)\, d\tilde{Q}(y)<\infty$, then $\hat{\ell }|\hat{Q}|^2$, $\hat{\ell }|\hat{\tilde{Q}}|^2$, $\hat{\ell }\hat{Q}\hat{\tilde{Q}}\in L^1(\mathbb {R}^d)$, and

2.11 $$\begin{aligned} \int _{\mathbb {R}^{2d}} \ell (x-y)\, dQ(x)\, dQ(y)&= (2\pi )^{-d} \int _{\mathbb {R}^{d}}\hat{\ell }(z) |\hat{Q}(z)|^2 dz, \end{aligned}$$

2.12 $$\begin{aligned} \int _{\mathbb {R}^{2d}} \ell (x-y)\, dQ(x)\, d\tilde{Q}(y)&= (2\pi )^{-d} \int _{\mathbb {R}^{d}}\hat{\ell }(z) \hat{Q}(z) \overline{ \hat{\tilde{Q}}(z) } \, dz. \end{aligned}$$

In particular, the identities (2.8), (2.9), (2.10) hold.

Now by the assumption $\hat{\ell }(z) \ge 0$, the two variance terms on the right hand side of (2.10) are nonnegative. Because the right hand side of (2.10) vanishes when $\nu =\delta _\mu$, i.e. $\mu _2=\mu \otimes \mu$, we conclude that $\mu _2=\mu \otimes \mu$ is a minimizer of the problem in (2.6), and hence (by Theorem 2.8) that $\gamma =\mu ^{\otimes \infty }=\mu \otimes \mu \otimes \cdots$ is a minimizer of (1.12). This establishes Theorem 1.2 (a).

Before proceeding with the proof of (b), let us note a corollary of the above arguments. By combining (2.4) and (2.10), we obtain:

#### **Corollary 2.11**

(Probabilistic interpretation of infinite-body optimal transport) Let $c(x,y)=\ell (x-y)$ be as in Theorem 1.2. If $\gamma \in {\mathcal {P}}_{sym}^\infty (\mathbb {R}^d)$, and if $\nu \in {\mathcal {P}}({\mathcal {P}}(\mathbb {R}^d))$ is the unique associated measure from Proposition 2.7 such that $\gamma =\int _{{\mathcal {P}}(\mathbb {R}^d)} Q^{\otimes \infty } d\nu (Q)$, then the functional $C_\infty$ introduced in (1.12) satisfies

$$\begin{aligned} C_\infty [\gamma ]&= \int _{\mathbb {R}^{2d}} \ell (x-y)\, \mu (dx) \, \mu (dy) \nonumber \\&+\, c_d \int _{\mathbb {R}^d} \hat{\ell }(z) \left( \mathrm{var}_{\nu (dQ)}Re(\hat{Q}(z)) + \mathrm{var}_{\nu (dQ)}Im(\hat{Q}(z)) \right) dz, \end{aligned}$$

where $\mu$ is the one-body marginal of $\gamma$ and where $c_d=(2\pi )^{-d}$.

It remains to show the uniqueness result (b). Suppose $\gamma$ is a minimizer of (1.12). By de Finetti’s theorem (2.7), there exists a probability measure $\nu \in {\mathcal {P}}({\mathcal {P}}(\mathbb {R}^d))$ such that

2.13 $$\begin{aligned} \gamma =\int _{{\mathcal {P}}(\mathbb {R}^d)} Q^{\otimes \infty } d\nu (Q). \end{aligned}$$

We have to show that $\nu$ is the Dirac mass $\delta _\mu$. By Theorem 2.8, the two-point marginal $\mu _2=\int _{{\mathcal {P}}(\mathbb {R}^d)} Q \otimes Q d\nu (Q)$ is a minimizer of the problem in (2.6). By (1.17), and (2.10), it follows that the right hand side of (2.10) is zero, i.e.

2.14 $$\begin{aligned} \int _{{\mathcal {P}}(\mathbb {R}^d)} |\hat{Q}(z)|^2 d\nu (Q) - \left| \int _{{\mathcal {P}}(\mathbb {R}^d)} \hat{Q}(z)\, d\nu (Q)\right| ^2 = 0\quad \text{ for } \text{ Lebesgue-a.e. }\, z\in \mathbb {R}^d. \end{aligned}$$

Because the left hand side equals $\int _{{\mathcal {P}}(\mathbb {R}^d)} | \hat{Q}(z) - \int _{{\mathcal {P}}(\mathbb {R}^d)} \hat{Q}(z)\, d\nu (Q)|^2 d\nu (Q)$, (2.14) holds if and only if

$$\begin{aligned} \hat{Q}(z) = \int _{{\mathcal {P}}(\mathbb {R}^d)} \hat{Q}(z) \, d\nu (Q)\quad \text{ for } \nu -\text{ a.e. }\, Q\in {\mathcal {P}}(\mathbb {R}^d). \end{aligned}$$

Therefore, by the injectivity of the Fourier transform as a map from ${\mathcal {P}}(\mathbb {R}^d)$ to $C_b(\mathbb {R}^d)$,

$$\begin{aligned} Q = \int _{{\mathcal {P}}(\mathbb {R}^d)} Q \, d\nu (Q) \quad \text{ for } \nu -\text{ a.e. }\, Q\in {\mathcal {P}}(\mathbb {R}^d). \end{aligned}$$

In other words, $\nu$ must be a Dirac mass (at $\mu$, to satisfy the margial constraint). Substitution into (2.13) shows that $\gamma$ is the independent measure (1.16). The proof of Theorem 1.2 is complete.

#### *Remark 2.12*

(a) The proof of Theorem 1.2 relies heavily on the positivity of the Fourier transform of $\ell$, and indeed the conclusion can fail dramatically in the absence of this condition, as shown by the following example.

#### *Example 2.13*

Let $\ell$ be any cost which is zero at $z=0$ and strictly positive elsewhere. Prototypical are

(i) the quadratic cost
  $$\begin{aligned} \ell (z)=|z|^2, \end{aligned}$$
  in which case (1.12) corresponds to the infinite marginal limit of the problem studied by Gangbo and Swiech in [32], in the special case of equal marginals; physically, one has replaced the repulsive Coulomb interactions by attractive harmonic oscillator-type interactions;
(ii) the smoothly truncated quadratic cost
  $$\begin{aligned} \ell (z) = e^{-|z|^2/2\sigma ^2} - e^{-\sigma ^2|z|^2/2}, \quad \sigma >1, \end{aligned}$$
  which behaves like $|z|^2$ near $z=0$ (so that (1.13) behaves like the quadratic OT problem (i) for marginals supported near $0$). Note that (ii) satisfies all assumptions of Theorem 1.2 except positivity of the Fourier transform $\hat{\ell }$ (note that $\hat{\ell }(k) = (\sqrt{2\pi }\sigma )^d e^{-\sigma ^2|k|^2/2} - (\sqrt{2\pi }/\sigma )^d e^{-|k|^2/2\sigma ^2}$).

It is clear that the probability measure $\gamma := (Id,Id,\ldots )_{\#}\mu$ (or, in physics notation, $\gamma (x_1,x_2,\ldots )=\mu (x_1)\delta _{x_1}(x_2)\delta _{x_1}(x_3)\cdots$) on $(\mathbb {R}^d)^\infty$ satisfies

$$\begin{aligned} C_\infty [\gamma ] = \int _{\mathbb {R}^{2d}}c(x,y) d\mu _2(x,y) =0, \end{aligned}$$

where $\mu _2$ is the 2-point marginal of $\gamma$. This is because $\mu _2=(Id,Id)_{\#}\mu$ (or, in physics notation, $\mu _2(x,y)=\mu (x)\delta _{x}(y)$) is concentrated on the diagonal $x=y$, where $c(x,y)=|x-y|^2=0$. Since trivially $C_\infty \ge 0$, the above $\gamma$ is a minimizer. However, by the positivity of $c(x,y)$off the diagonal, the independent measure $\mu \otimes \mu \otimes \cdots$ is not a minimizer except in the trivial case when $\mu =\delta _x$ for some $x\in \mathbb {R}^d$.

(b) Examples of cost functions which satisfy the assumptions of Theorem 1.2 include:

(i) the Gaussian cost $\ell (z) = e^{-|z|^2/2\sigma ^2},\sigma \ne 0$, with Fourier transform $\hat{\ell }(k) = (\sqrt{2\pi }\sigma )^d e^{-\sigma ^2|k|^2/2}$, and the exponential cost $\ell (z)=e^{-a|z|},z\in \mathbb {R}^d,a>0$, with Fourier transform $\hat{\ell }(k)=(2\pi )^dc(d)a/(a^2+k^2)^{\frac{d+1}{2}}, k\in \mathbb {R}^d$, where $c(d)=\Gamma ((d+1)/2)/\pi ^{\frac{d+1}{2}}$, with $\Gamma$ being the gamma function. Both satisfy the assumptions of Theorem 1.2(a).
(ii) Let $\lambda ,\beta >0, \beta >d/2,$ and let $l_{\lambda , \beta }(z)=\left( |z|^2+\lambda ^2\right) ^{-\beta }, z\in \mathbb {R}^d$ ($l_{\lambda ,\beta }$ are the *inverse multiquadric functions*, widely used in statistics and in machine learning). By Theorem 6.13 from [66], $l_{\lambda ,\beta }$ has as Fourier transform
  $$\begin{aligned} {\hat{l}}_{\lambda ,\beta }(k)=c(\lambda ,\beta ,d)|k|^{\beta -d/2} K_{\beta -d/2}(|k|),\quad k\in \mathbb {R}^d, \end{aligned}$$
  where $c(\lambda ,\beta ,d)>0$ depends only on $\alpha ,\beta$ and $d$, and where $K_{\beta -d/2}\ge 0$ is the modified Bessel function of second kind of order $\beta -d/2$. Moreover, $l_{\lambda ,\beta } \in L^1(\mathbb {R}^d)\cap C_b(\mathbb {R}^d)$ so Theorem 1.2(a) applies.
(iii) A natural extension to the Coulomb cost example from Theorem 1.2(b) is the so-called *screened Coulomb potential* (also known in physics as Yukawa potential). Set, for each $\epsilon >0$, $c_{\epsilon }(x,y) =\frac{e^{-\epsilon |x-y|}}{|x-y|}, x,y\in \mathbb {R}^3$. Since $c_{\epsilon }(x,y)\le c(x,y)=\frac{1}{|x-y|}$ for all $\epsilon \ge 0$ and all $x,y\in \mathbb {R}^3$, and since (1.15) holds for the Coulomb cost $c$, we have for all $\mu \in L^1(\mathbb {R}^3)\cap L^3(\mathbb {R}^3)$
  2.15 $$\begin{aligned} \int _{\mathbb {R}^{6}} \frac{e^{-\epsilon |x-y|}}{|x-y|} \, \mu (x)\mu (y)dx dy<\infty ,~~\text{ for } \text{ all }~\epsilon >0. \end{aligned}$$
  We note that $\ell _{\epsilon }(x): =\frac{e^{-\epsilon |x|}}{|x|}, x\in \mathbb {R}^3,$ is in $L^1(\mathbb {R}^3)$, is continuous on $(0,\infty )$, and has Fourier transform $\hat{\ell }_\epsilon (k)=\frac{4\pi }{|k|^2+\epsilon ^2}>0, k\in \mathbb {R}^d$ (see, for example, [43] for a proof). Even though $\ell _\epsilon$ is not bounded for any $\epsilon >0$, the result can be proven for this cost by a straightforward adaptation of our argument for the Coulomb cost.
(iv) Various constraints ensuring that the Fourier transform of a function is positive have been derived for example in [36].

(c) A representation similar to the Finetti representation (2.5) but with $\nu \in {\mathcal {P}}({\mathcal {P}}(\mathbb {R}^d))$ replaced by a *signed* measure has been established in [44]. Such a representation would allow us to derive (2.6), but—due to the lack of sign information—does not allow to conclude that the independent measure is optimal in the finite-$N$ case. Indeed, in the special case of marginals supported on two points it follows from the analysis in [27] that the independent measure is not minimizing for *any* $N$. For more general densities and cost functions, it follows from Proposition 3.6 below that the independent measure is not minimizing for any N.

(d) As a corollary of our analysis, we recover the following interesting result from [39]: if $(X_n)_{n\ge 1}$ is an infinite sequence of exchangeable random variables in $\mathbb {R}^d$ such that $(X_n)_{n\ge 1}$ are pairwise independent (i.e., the joint distribution of any $(X_i,X_j)$ is a product of the distributions of $X_i$ and $X_j$), they are mutually independent.

Before we prove the above, we give the definition of exchangeable random variables. Formally, a * finite (respectively infinite) exchangeable sequence of random variables* is a finite (respectively infinite) sequence $X_1, X_2, X_3, \ldots$ of random variables such that for any finite permutation $\tau$ of the indices $1, 2, 3, \ldots$ (the permutation acts on only finitely many indices, with the rest fixed), the joint probability distribution of the permuted sequence

$$\begin{aligned} X_{\tau (1)}, X_{\tau (2)}, X_{\tau (3)}, \dots \end{aligned}$$

is the same as the joint probability distribution of the original sequence. Let $\gamma$ be the joint distribution of the infinite sequence $(X_1,X_2,\ldots )$, let $\mu _2$ be the distribution of $(X_1,X_2)$, and let $\mu$ be the distribution of $X_1$. By the assumption of pairwise independence, $\mu _2=\mu \otimes \mu$. Hence, fixing for instance the cost $\ell (z)=e^{-|z|^2}$ and combining Eq. (1.17) and Lemma 2.4, it follows that $\gamma$ is a minimizer of (1.12). But the uniqueness result of Theorem 1.2(b) implies that the only minimizer of (1.12) is the independent measure $\mu \otimes \mu \otimes \cdots$. Thus $\gamma =\mu \otimes \mu \otimes \cdots$, as was to be shown.

Note that for $N<\infty$, pairwise independence does not imply mutual independence. One of the first counter-examples for $N<\infty$ was provided in [4]; for further counter-examples see e.g. [21].

(e) We note that weakening even slightly the assumption of exchangeability of the measure may destroy uniqueness of the minimizer of (1.12). To prove this, we apply for example the results from [41] or from [8]. Therein, various examples are constructed of infinite stationary sequences $(X_n)_{n\ge 1}$ of random variables in $\mathbb {R}^d$ such that $(X_n)_{n\ge 1}$ are pairwise independent, with mean $0$ and finite second moments, but which do not satisfy the central limit theorem. This implies that in these particular cases $(X_n)_{n\ge 1}$ are not mutually independent.

## Connection between the N-body OT problem and the infinite-body OT problem

We will establish in this section the relationship between the infinite-body optimal transport problem (1.12) and the corresponding $N$-body optimal transportation problem (1.18), as stated in our second main result Theorem 1.3. We recall first from (1.19) the optimal cost of the $N$-body problem per particle pair, given for all $N\in {\mathbb {N}}$, $N\ge 2$ by

$$\begin{aligned} {F}^{OT}_N[\mu ] := \frac{1}{{N\atopwithdelims ()2}} \inf _{\gamma \in {\mathcal {P}}_{sym}^N(\mathbb {R}^{d}),\gamma \,\mapsto \mu }{C}_N[\gamma ]. \end{aligned}$$

Moreover, analogously to Lemma 2.4 (see also Theorem III.3 in [27]) we have

3.1 $$\begin{aligned} {F}^{OT}_{N}[\mu ] = \inf \Bigl \{\int _{\mathbb {R}^{2d}} c(x,y) \, d\mu _2(x,y) \; \Big | \; \mu _2\in {\mathcal {P}}^2_{sym}(\mathbb {R}^{d}), \, \mu _2\mapsto \mu , \, \mu _2 \text{ is } \text{ N-representable } \Bigr \}. \end{aligned}$$

This representation will be used in the proof of Theorem 1.3.

We first note the following existence result for (3.1):

### **Proposition 3.1**

Let $c_N\, : (\mathbb {R}^d)^N \rightarrow {\mathbb {R}}_+\cup \{\infty \}$ be defined as in (1.10), with $c$ lower semi-continuous. Then there exists at least one solution $\gamma _N$ to (1.18), and at least one solution $\mu _{2,N}\in {\mathcal {P}}_{sym}^2(\mathbb {R}^{d})$ to the minimization problem in (3.1).

### *Proof*

The proof follows from a standard compactness argument, similar to those found in [65], combined with the fact that a non symmetric measure $\gamma$ on $\mathbb {R}^{Nd}$ may be symmetrized without changing the total cost $C_N[\gamma ]$, due to the linearity of the functional and the constraints, and the symmetry of $c$. $\square$

To establish (1.20), we will use the following result which allows us to approximate $N$-representable measures by infinitely representable ones. The result is actually a translation of Theorem $13$ in [22] from the language of random variables into that of probability measures and explains why De Finetti’s Theorem holds exactly for $N=\infty$ but only approximately for $N<\infty$. For purposes of simplicity and completeness, unlike [22] we limit ourselves to euclidean spaces, and include a proof.

### **Proposition 3.2**

Let $\gamma _N\in {\mathcal {P}}_{sym}^N(\mathbb {R}^{d})$. Then there exists an infinitely representable measure ${\mathbb {P}}_{2,\gamma _N}\in \mathcal{P}_{sym}^2(\mathbb {R}^{d})$ such that

3.2 $$\begin{aligned} ||\gamma _2-{\mathbb {P}}_{2,\gamma _N}||\le \frac{1}{N}\qquad \text{ and }\qquad \gamma _1={\mathbb {P}}_{1,\gamma _N}. \end{aligned}$$

For $1\le k\le N$, we denoted in (3.2) by $\gamma _k$ the canonical projection of $\gamma _N$ on $\mathcal{P}_{sym}^k(\mathbb {R}^{d})$ (that is, $\gamma _k\in {\mathcal {P}}^k_{sym}(\mathbb {R}^{d})$ is a marginal of $\gamma _N)$, and by $||\gamma _k-\mathbb {P}_{k,\nu }||$ the total variation distance, that is,

$$\begin{aligned} ||\gamma _k-{\mathbb {P}}_{k,\gamma _N}||:=\sup _{\begin{array}{c} \{f:\mathbb {R}^d\rightarrow \mathbb {R}, \\ f~\text{ measurable },~ |f|\le 1\} \end{array}}|\gamma _k(f)-\mathbb {P}_{k,\nu }(f)|. \end{aligned}$$

### *Proof*

To prove (3.2), let us define for each $k\ge 1$ the measure ${\mathbb {P}}_{k,\gamma _N}\in \mathcal{P}(\mathbb {R}^{kd})$ by

3.3 $$\begin{aligned} {\mathbb {P}}_{k,\gamma _N}(A_k):=\int _{\mathbb {R}^{Nd}}\left( \frac{{\delta }_{\omega _1}+{\delta }_{\omega _2}+\cdots +{\delta }_{\omega _N}}{N}\right) ^{\otimes k}(A_k)\,d\gamma _N(\omega ),~~\text{ for } \text{ all }~A_k\in \mathbb {R}^{kd}. \end{aligned}$$

By Kolmogorov’s extension theorem, ${\mathbb {P}}_{k,\gamma _N}$ can be extended to an infinite-dimensional symmetric measure ${\mathbb {P}}_{\infty ,\nu }$ in ${\mathcal {P}}_{sym}^\infty (\mathbb {R}^d)$, which has ${\mathbb {P}}_{k,\gamma _N}$ as marginal for each $k\ge 1$. Moreover, for all $A_2\in {\mathbb {R}}^{2d}$ we obtain from (3.3) that

$$\begin{aligned} {\mathbb {P}}_{2,\gamma _N}(A_2)&= \int _{\mathbb {R}^{Nd}}\left( \frac{{\delta }_{\omega _1}+{\delta }_{\omega _2}+\cdots +{\delta }_{\omega _N}}{N}\right) ^{\otimes 2}(A_2)\,d\gamma _N(\omega )\\&= \frac{N^2-N}{N^2}\gamma _2(A_2)+\frac{1}{N}\gamma _1(\{\omega _1:(\omega _1,\omega _1)\in A_2\} ), \end{aligned}$$

and therefore

$$\begin{aligned} \left| \gamma _2(A_2)- {\mathbb {P}}_{2,\gamma _N}(A_2) \right| =\frac{1}{N}\left| \gamma _2(A_2)-\gamma _1(\omega _1:(\omega _1,\omega _1)\in A_2)\right| \le \frac{1}{N}. \end{aligned}$$

$$\square $$

We will use this result directly to easily establish Theorem 1.3 part (i). For part (ii), we will need the following intermediate Lemma.

### **Lemma 3.3**

- Let $\mu \in {\mathcal {P}}(\mathbb {R}^d)$ and let $\left( \mu _{2,N}\right) _{N\ge 2}$ be a sequence of symmetric probability measures on $\mathbb {R}^{2d}$ such that $\mu _{2,N}\mapsto \mu$ and $\mu _{2,N}$ is $N$-representable for all $N.$ If $\mu _{2,N}$ converges weakly to some symmetric probability measure $\mu _2$ on $\mathbb {R}^{2d}$, then $\mu _2$ is infinitely representable.
- A symmetric probability measure $\mu _2$ on $\mathbb {R}^{3d}$ is infinitely representable if and only if it is $N$-representable for all $N\ge 2$.

### *Proof*

First we deal with part (a). Proposition 3.2 yields a sequence of infinitely representable measures $({\mathbb {P}}_{2,N}\in {\mathcal {P}}_{sym}^2(\mathbb {R}^d)))_{N\ge 2}$ converging weakly to $\mu _2$. By definition, for each ${\mathbb {P}}_{2,N}$ there exists $\gamma ^N\in {\mathcal {P}}_{sym}^\infty (\mathbb {R}^d)$ such that $\gamma ^N\mapsto {\mathbb {P}}_{2,N}$. By the same reasoning as in Theorem 2.9(b), the measures $\gamma ^N\in {\mathcal {P}}_{sym}^\infty (\mathbb {R}^d), N\ge 2,$ all lie in a tight set of $(\mathbb {R}^d)^\infty$, so by Prokhorov’s theorem we can extract a further subsequence, still denoted by $(\gamma ^N)_{N\in \mathbb {N}}$ for simplicity, which converges weakly to some $\gamma ^{\lim }\in {\mathcal {P}}((\mathbb {R}^d)^\infty )$, $\gamma ^{\lim }\mapsto \mu _2$. We recall now that the class ${\mathcal {P}}_{sym}^\infty (\mathbb {R}^d)$ of infinite-dimensional symmetric probability measures is closed under weak convergence, therefore $\gamma ^{\lim }\in {\mathcal {P}}_{sym}^\infty (\mathbb {R}^d)$. It follows that $\mu _2$ is infinitely representable.

Next we prove (b). It is clear that an infinitely representable measure is $N$-representable for all $N\ge 2$. On the other hand, if $\mu _2$ is $N$-representable for all $N\ge 2$, the result follows from assertion (a) by taking $\mu _{2,N}\equiv \mu _2$ for all $N\ge 2$. $\square$

### *Proof of Theorem 1.3*

We first prove part (i) (the bounded costs case) directly from Proposition 3.2. Letting $\mu _{2,N}$ solve (3.1), we have by Proposition 3.2 an infinitely representable $\mu _{2,\infty }$ with 1-body marginal $\mu$ such that $||\mu _{2,N}-\mu _{2,\infty }|| \le \frac{1}{N}$. Therefore

3.4 $$\begin{aligned} F_N^{OT}[\mu ]&= \int _{\mathbb {R}^{2d}}c(x,y)d\mu _{2,N}\end{aligned}$$

3.5 $$\begin{aligned}&\ge \int _{\mathbb {R}^{2d}}c(x,y)d\mu _{2,\infty } -\frac{||c||_\infty }{N}\end{aligned}$$

3.6 $$\begin{aligned}&\ge F_{\infty }^{OT}[\mu ] -\frac{||c||_\infty }{N}. \end{aligned}$$

Noting that $F_N^{OT}[\mu ] \le F_{\infty }^{OT}[\mu ]$ and taking the limit in the above inequality yields the result.

We will prove next assertion (ii). Let $\mu _{2,N}\in {\mathcal {P}}_{sym}^2(\mathbb {R}^d), N\ge 2,$ where $\mu _{2,N}$ is $N$-representable, solve (3.1). By the tightness of the set of symmetric measures on $\mathbb {R}^{2d}$ with common marginal $\mu$ and by Prokhorov’s theorem, we can, after passing to a subsequence, assume that $\mu _{2,N}$ converges weakly to some measure $\mu _2\in {\mathcal {P}}_{sym}^2(\mathbb {R}^d)$ whose marginal is also $\mu$. By Lemma 3.3, it immediately follows that $\mu _2$ is infinitely representable.

By lower semi-continuity of $c$, we therefore have

3.7 $$\begin{aligned} \liminf _{N\rightarrow \infty }F_N^{OT}[\mu ] =\liminf _{N\rightarrow \infty } \int _{\mathbb {R}^{2d}}c(x,y)d\mu _{2,N} \ge \int _{\mathbb {R}^{2d}}c(x,y)d\mu _{2} \ge F_{\infty }^{OT}[\mu ]. \end{aligned}$$

As we clearly have $F_N^{OT}[\mu ] \le F_{\infty }^{OT}[\mu ]$ for each $N$, this implies the desired result. $\square$

### *Remark 3.4*

- We note here that the proof in fact yields that any convergent subsequence of optimal $\mu _{2,N}$ in the $N$-body problem converges to a solution to the infinite body problem. Whenever the minimizer $\mu _{2,\infty }$ in the infinite body problem is unique (for example, under the conditions in Theorem 1.2 part (ii)), this implies that the $\mu _{2,N}$ converge to $\mu _{2,\infty }$. For bounded costs, the proof also yields a bound on the rate of convergence of $\frac{||c||_\infty }{N}$.
- Theorem $13$ from [22] proves the following: Let $\gamma _N\in {\mathcal {P}}_{sym}^N(\mathbb {R}^{d})$. Then there exists a measure $\nu$ on the set of probability measures on $\mathcal{P}(\mathbb {R}^d)$, such that
  3.8 $$\begin{aligned} ||\gamma _k-{\mathbb {P}}_{k,\nu }||\le \frac{k(k-1)}{N}\quad \text{ for } \text{ all }~1\le k\le N. \end{aligned}$$
  For some particular cases of marginals $\gamma _1$ the bounds in (3.8) have been improved in [23].

Next we point out a variant of our result in Corollary 1.4 on the inhomogeneous high-density limit of the SCE functional introduced in (1.1), (1.2). By Eq. (1.21) together with the characterization (3.1) of $F^{OT}_N$ as an infimum over representable pair measures (or alternatively Theorem III.3 in [27]), we have

3.9 $$\begin{aligned}&{V}_{ee}^{SCE}[\rho ] \nonumber \\&\quad = {N\atopwithdelims ()2} \inf \left\{ \int _{\mathbb {R}^{6}} \frac{1}{|x-y|} \, d\mu _2(x,y) \; \Big | \; \mu _2\in {\mathcal {P}}^2_{sym}(\mathbb {R}^{3}), \, \mu _2\mapsto \rho /N, \, \mu _2 \text{ N-representable } \right\} ,\nonumber \\ \end{aligned}$$

where $\rho$ is any integrable nonnegative function on $\mathbb {R}^3$ with $\int _{\mathbb {R}^3}\rho =N$. This formula suggests a natural hierarchy of approximations as introduced in [27]: for $k=2,3,\ldots$ we define

3.10 $$\begin{aligned}&{V}_{ee}^{SCE,k}[\rho ] \nonumber \\&\quad :={N\atopwithdelims ()2} \inf \left\{ \int _{\mathbb {R}^{6}} \frac{1}{|x-y|} \, d\mu _2(x,y) \; \Big | \; \mu _2\in {\mathcal {P}}_{sym}^2(\mathbb {R}^{3}), \, \mu _2\mapsto \rho /N, \, \mu _2 \text{ k-representable } \right\} .\nonumber \\ \end{aligned}$$

That is, we replace the requirement that $\mu _2$ is $N$- representable by the modified requirement that it is $k$-representable. Because $k$-representability becomes a stronger and stronger condition as $k$ increases, we have the following chain of inequalities

$$\begin{aligned} {V}_{ee}^{SCE,2}[\rho ]\le \cdots \le V_{ee}^{SCE,3}[\rho ]\le \cdots \le {V}_{ee}^{SCE,N}[\rho ] = V_{ee}^{SCE}[\rho ]\le {V}_{ee}^{SCE,N+1}[\rho ]\le \cdots \,. \end{aligned}$$

The functionals ${V}_{ee}^{SCE,k}$ can be thought of as reduced models for the energy of strongly correlated electrons which take into account $k$- body correlations.

### **Corollary 3.5**

Assume that $\rho \in L^1(\mathbb {R}^3)$, $\rho \ge 0$, $\int _{\mathbb {R}^3}\rho = N$ for some natural number $N\ge 2$. Then

3.11 $$\begin{aligned} \lim _{k\rightarrow \infty } V_{ee}^{SCE,k}[\rho ] = \frac{1}{2} \left( 1-\frac{1}{N}\right) \int _{R^6}\frac{1}{|x-y|}\rho (x)\rho (y) dx dy. \end{aligned}$$

Physically, the factor $1-1/N$ is a self-interaction correction, and the right hand side of (3.11) is a self-interaction corrected mean field energy. Thus the approximation via density representability of infinite order remembers that there are only ${N\atopwithdelims ()2}$ interaction terms, not $N^2/2$.

### *Proof*

By the Definition (3.10), for any $\rho$ as above we have

$$\begin{aligned} V_{ee}^{SCE,k}[\rho ] = {N\atopwithdelims ()2} F_k^{OT}[\rho /N], \end{aligned}$$

that is to say, up to scaling factors $V_{ee}^{SCE,k}[\rho ]$ is the optimal cost of a $k$-body optimal transport problem. By Theorems 1.3 and 1.2, the right hand side converges to

$$\begin{aligned} {N\atopwithdelims ()2} \int _{\mathbb {R}^6} \frac{ \frac{\rho (x)}{N} \, \frac{\rho (y)}{N} }{|x-y|} \, dx \, dy \end{aligned}$$

as $k\rightarrow \infty$. This establishes the corollary. $\square$

Finally we note that, in contrast to the $N=\infty$ case, minimizers of the $N$-body optimal transport problem are typically not given by the mean field measure for any $N<\infty$.

### **Proposition 3.6**

Let $c_N:(\mathbb {R}^d)^N \rightarrow {\mathbb {R}}_+\cup \{\infty \}$ be defined as in (1.10). Assume that there is some point $x=(x_1,x_2,\ldots ,x_N) \in \mathbb {R}^{Nd}$ such that $c_N$ is $C^2$ near $x$, $D^2_{x_ix_j}c(x) \ne 0$ for some $i \ne j$, and the measure $\mu$ has positive density near each $x_i \in \mathbb {R}^d$. Then the product measure $\mu \otimes \mu$ on $\mathbb {R}^{2d}$ is not optimal for the $2$-body optimal transport problem with $N$-representability constraint (3.1), for any $N < \infty$.

Note that for the Coulomb cost, the conditions on the cost hold for any $x=(x_1,x_2,\ldots ,x_N)$ away from the diagonal; that is, for any $x$ such that $x_i \ne x_j$ for all $i \ne j$.

### *Proof*

Fix $N<\infty$. The proof is by contradiction; assume that the product measure $\mu ^{\otimes 2}$ on $\mathbb {R}^{2d}$ is optimal for (3.1). Then the product measure $\mu ^{\otimes N}$ on $\mathbb {R}^d \times \mathbb {R}^d \times \cdots \times \mathbb {R}^d$ must be optimal for the $N$-body optimal transport formulation of the problem (1.12). It is clear that the support of the product measure has full Hausdorff dimension $dN$ near the point $x$. On the other hand, Theorem 2.3 from [51] implies that for any optimizer $\gamma$, for some neighbourhood $U$ of $x$, the dimension of the $supp(\gamma ) \cap U$ is no more than $\lambda _0+\lambda _-$, where $supp(\gamma )$ is the support of $\gamma$, and $\lambda _+, \lambda _-$ and $\lambda _0$ are respectively the number of positive, negative and zero eigenvalues of the off-diagonal part of the Hessian

3.12 $$\begin{aligned} \qquad G= \begin{bmatrix} 0&\quad D^{2}_{x_1x_2}c&\quad D^{2}_{x_1x_3}c&\quad \ldots&\quad D^{2}_{x_1x_N}c \\ D^{2}_{x_2x_1}c&\quad 0&\quad D^{2}_{x_2x_3}c&\quad \ldots&\quad D^{2}_{x_2x_N}c \\ D^{2}_{x_3x_1}c&\quad D^{2}_{x_3x_2}c&\quad 0&\quad \ldots&\quad D^{2}_{x_3x_N}c \\ \cdots&\quad \cdots&\quad \cdots&\quad \cdots&\quad \cdots ,\\ D^{2}_{x_Nx_1}c&\quad D^{2}_{x_Nx_2}c&\quad D^{2}_{x_Nx_3}c&\quad \ldots&\quad 0 \end{bmatrix} \end{aligned}$$

evaluated at $x$. Therefore, if $\mu ^{\otimes N}$ is optimal, $G$ must have no positive eigenvalues and therefore must be negative semi-definite. This is clearly not true; as $D^{2}_{x_ix_j}c \ne 0$, we can choose $u, v \in \mathbb {R}^d$ such that $u \cdot D^{2}_{x_ix_j}c\cdot v^T >0$. Then

$$\begin{aligned}&[0,\ldots ,0,u,0,\ldots ,0,v,0,\ldots ,0] \cdot G \cdot [0,\ldots ,0,u,0,\ldots ,0,v, ,0,\ldots ,0]^T \\&\quad = v \cdot D^{2}_{x_jx_i}c\cdot u^T + u\cdot D^{2}_{x_ix_j}c\cdot v^T \\&\quad = 2u\cdot D^{2}_{x_ix_j}c\cdot v^T\\&\quad > 0, \end{aligned}$$

contradicting the negative definiteness of $G$. $\square$

## Conclusions

Mean field approximations that reduce complicated many-body interactions to interactions of each particle with a collective mean field are ubiquitous in many areas of physics such as quantum mechanics, statistical mechanics, electromagnetism, and continuum mechanics, as well as in other fields such as mathematical biology, probability theory, or game theory.

Motivated by questions in many-electron quantum mechanics, we have presented a novel and quite general mathematical picture of how mean field approximations are rigorously related to underlying many-body interactions. Namely, for interactions with positive Fourier transform they emerge as the unique solution to a naturally associated infinite-body optimal transport problem.

## Appendix

Here we prove the result stated in Lemma 2.10 that a well known formula from Fourier transform calculus on $\mathbb {R}^d$ remains valid for integrals involving two probability measures and a cost function such as the Coulomb cost.

The formula would be straightforward if the probability measures and the cost function belonged to $L^1(\mathbb {R}^d)$. The generalization to arbitrary probability measures was essential in the proof of our main result that the solution to infinite-body optimal transport problems for costs with positive Fourier transform is the independent product measure. We note that the generalization is needed even in the case of smooth marginals, since general probability measures always appear in the de Finetti representation (2.5) of trial measures.

### *Proof of Lemma 2.10*

First we deal with the case $\ell \in C_b(\mathbb {R}^d)\cap L^1(\mathbb {R}^d)$. We begin by proving (2.11). The idea is to regularize $Q$. Let $G_\varepsilon$ be a Gaussian with standard deviation $\varepsilon$, i.e. $G_\varepsilon (x)=(2\pi \varepsilon ^2)^{-d/2} e^{-|x|^2/2\epsilon ^2}$. Then $\widehat{G_\varepsilon }(k) = e^{-\varepsilon |k|^2/2}$. By inspection, $\hat{G_\varepsilon }$ converges *monotonically* to $1$ as $\varepsilon \rightarrow 0$. The monotonicity of this convergence is actually needed in the argument below.

Now for any given probability measure $Q$ on $\mathbb {R}^d$, let $Q_\varepsilon$ be the regularization $Q_\varepsilon (x)=(G_\varepsilon * Q)(x) = \int _{\mathbb {R}^d} G_\varepsilon (x-y)\, dQ(y)$. Then $Q_\varepsilon \in L^1(\mathbb {R}^d)\cap L^\infty (\mathbb {R}^d)$; in particular $Q_\varepsilon \in L^2$. Next we claim that $\ell *Q_\varepsilon \in L^2(\mathbb {R}^d)$. This is because $\ell *Q_\varepsilon$ is, as a convolution of two $L^1$ functions, in $L^1$, and also, as a convolution of an $L^1$ and an $L^\infty$ function, in $L^\infty$.

Since $\ell$ and $Q_\varepsilon$ are in $L^1(\mathbb {R}^d)$, it is straightforward from the definition of the Fourier transform on $L^1$ as a convergent integral that $\widehat{\ell * Q_\varepsilon } = \widehat{\ell } \, \widehat{Q_\varepsilon }$. It follows that formula (2.11) is valid for the regularized measure $Q_\varepsilon$, i.e.

4.1 $$\begin{aligned} \int _{\mathbb {R}^{2d}} \ell (x-y)\, dQ_\varepsilon (x) dQ_\varepsilon (y) = (2\pi )^{-d} \int _{\mathbb {R}^d} \hat{\ell }(z) \, |\hat{Q_\varepsilon }(z)|^2 dz. \end{aligned}$$

It remains to pass to the limit $\varepsilon \rightarrow 0$. Since $Q_\varepsilon \rightharpoonup Q$ weakly (that is to say $\int _{\mathbb {R}^d} \varphi Q_\varepsilon \rightarrow \int _{\mathbb {R}^d} \varphi \, dQ$ for all $\varphi$ belonging to the space $C_b(\mathbb {R}^d)$ of bounded continuous functions), we have $Q_\varepsilon \otimes Q_\varepsilon \rightharpoonup Q\otimes Q$, and since the function $(x,y)\mapsto \ell (x,y) \in C_b(\mathbb {R}^{2d})$ we infer that the left hand side of (4.1) converges to the left hand side of (2.11). Since $\widehat{Q_\varepsilon } = \widehat{G_\varepsilon } \, \widehat{Q}$, $\widehat{\ell }\ge 0$, and $\widehat{G_\varepsilon }$ converges monotonically to $1$, the integrand on the right hand side of (4.1), $\widehat{\ell } |\widehat{Q_\varepsilon }|^2 = \hat{\ell }|\widehat{G_\varepsilon }|^2|\widehat{Q}|^2$, converges monotonically to $\widehat{\ell }$. Hence by monotone convergence, the right hand side of (4.1) tends to that of (2.11), establishing (2.11).

It remains to prove (2.12). Analogously to the proof of (2.11) we obtain

4.2 $$\begin{aligned} \int _{\mathbb {R}^{2d}} \ell (x-y) \, Q_\varepsilon (x) \, \tilde{Q}_\varepsilon (y) \, dx \, dy = (2\pi )^{-d} \int _{\mathbb {R}^d} \widehat{\ell } \widehat{Q_\varepsilon } \overline{\widehat{Q_\varepsilon }} \end{aligned}$$

as well as the fact that the left hand side tends to the left hand side of (2.12) as $\varepsilon \rightarrow 0$. The argument for passing to the limit on the right hand side no longer works, since now the integrand is not in general nonnegative. Instead we use that by the assumption of finiteness of $\int \ell (x-y)dQ(x)dQ(y)$ and $\int \ell (x-y)d\tilde{Q}(x)d\tilde{Q}(y)$ and by (2.11), $\hat{\ell }|\widehat{Q}|^2$ and $\hat{\ell }|\widehat{\tilde{Q}}|^2$ are in $L^1(\mathbb {R}^d)$. This together with the pointwise estimate

$$\begin{aligned} | \widehat{Q_\varepsilon } \, \widehat{\tilde{Q}_\varepsilon } | \le \frac{1}{2} \left( |\widehat{Q}|^2 + |\widehat{\tilde{Q}}|^2 \right) \end{aligned}$$

(which relies on $\widehat{Q_\varepsilon } = \widehat{G_\varepsilon }\widehat{Q}$ and $|\widehat{G_\varepsilon }|\le 1$) shows that the convergence $\widehat{\ell }\widehat{Q_\varepsilon }\widehat{\tilde{Q}_\varepsilon }\rightarrow \widehat{\ell }\widehat{Q}\widehat{\tilde{Q}}$ is dominated. Hence by the dominated convergence theorem the right hand side of (4.2) tends to that of (2.12) as $\varepsilon \rightarrow 0$. This completes the proof of Lemma 2.10 in the case $\ell \in C_b\cap L^1$.

It remains to deal with the Coulomb case $d=3$, $\ell (x)=\frac{1}{|x|}$. In this case the above proof does not work, for instance because weak convergence of the probability measure $Q_\varepsilon \otimes Q_\varepsilon$ is insufficient to pass to the limit in the left hand side of (4.1) due to the fact that $(x,y)\mapsto \ell (x,y)$ no longer belongs to the space $C_b$ of bounded continuous functions associated by duality. However the desired Fourier identities were established in [11], with passage to the limit in (4.1) being achieved with the help of Newton’s screening theorem. The latter is the special Coulombic property that for any continuous radially symmetric function $\varphi$ with compact support, $\varphi *1/|\cdot |=1/|\cdot |$ outside the support of $\varphi$ (or, physically speaking, the potential exerted by a radial charge distribution onto a point outside it is the same as that of the point charge obtained by placing all its mass at the center).

## References

[1] Ayers PW, Davidson ER (2006). Necessary conditions for the N-representability of pair distribution functions. Int. J. Quantum Chem..

[2] Aldous, D.: Exchangeability and related topics. Ecole d’Ete St Flour 1983. In: Proceedings of Lecture Notes in Mathematics, vol. 1117, pp. 1–198. Springer, New York (1985)

[3] Becke A (1993). Density-functional thermochemistry III: the role of exact exchange. J. Chem. Phys..

[4] Bernstein, S.N.: Theory of Probability. Gostechizat, Moscow-Leningrad, 4th edn. (in Russian) (1946)

[5] Billingsley, P.: Convergence of Probability Measures. Wiley, New York (1999)

[6] Bogachev, V.I.: Measure Theory, vol. 1. Springer, New York (2007)

[7] Borwein, J., Lewis, A.: Convex Analysis and Nonlinear Optimization: Theory and Examples, 2 edn. Springer, New York (2006)

[8] Bradley R (1989). A stationary, pairwise independent, absolutely regular sequence for which the central limit theorem fails. Prob. Theory Rel. Fields.

[9] Brenier, Y.: Decomposition polaire et rearrangement monotone des champs de vecteurs. C. R. Acad. Sci. Pair. Ser. I Math. **305**, 805–808 (1987)

[10] Buttazzo G, De Pascale L, Gori-Giorgi P (2012). Optimal transport formulation of electronic density-functional theory. Phys. Rev. A.

[11] Capet, S., Friesecke, G.: Minimum energy configurations of classical charges: large N asymptotics. Appl. Math. Res. Exp. (2009). doi:10.1093/amrx/abp002

[12] Carlier G (2003). On a class of multidimensional optimal transportation problems. J. Convex Anal..

[13] Carlier G, Nazaret B (2008). Optimal transportation for the determinant. ESAIM Control Optim. Calc. Var..

[14] Coleman, A.J., Yukalov, V.I.: Reduced density matrices: Coulson’s challenge. In: Proceedings of Lecture Notes in Chemistry, Springer, New York (2000)

[15] Colombo, M., Di Marino, S.: Equality between Monge and Kantorovich multimarginal problems with Coulomb cost, preprint (2013)

[16] Colombo, M., De Pascale, L., Di Marino, S.: Multimarginal optimal transport maps for 1-dimensional repulsive costs, preprint (2013)

[17] Cotar C, Friesecke G, Klüppelberg C (2013). Density functional theory and optimal transportation with Coulomb cost. Comm. Pure Appl. Math..

[18] Cotar, C., Friesecke, G., Klüppelberg, C.: Smoothing of transport plans with fixed marginals and rigorous semiclassical limit of the Hohenberg–Kohn functional, preprint (2013) 10.1063/1.4821351 24182006

[19] Davidson ER (1995). N-representability of the electron pair density. Chem. Phys. Lett..

[20] de Finetti B (1969). Sulla proseguibilitá di processi aleatori scambiabili. Rend. Matem. Trieste.

[21] Derriennic, Y., Klopotowski, A.: On Bernstein’s example of three pairwise independent random variables. Sankhya Indian J. Stat. **62A**(3), 318–330 (2000)

[22] Diaconis P, Freedman D (1980). Finite Exchangeable Sequences. Ann. Probab..

[23] Diaconis P, Freedman D (1987). A dozen de Finetti-style results in search of a theory. Ann. Inst. H. Poincar Probab. Stat..

[24] Dunford N, Schwartz JT (1958). Linear Operators.

[25] Fiolhais, C., Noqueira, F., Marques, M.: (eds.) A primer in density functional theory. In: Proceedings of Lecture Notes in Physics, vol. 620. Springer, Berlin, Heidelberg, New York (2003)

[26] Friesecke G (2003). The multiconfiguration equations for atoms and molecules: charge quantization and existence of solutions. Arch. Rat. Mech. Anal..

[27] Friesecke, G., Mendl, C. Pass, B., Cotar, C., Klüppelberg, C.: N-density representability and the optimal transport limit of the Hohenberg–Kohn functional. J. Chem. Phys. **139**(16), 164109 (2013) 10.1063/1.4821351 24182006

[28] Fristedt BE, Gray LF (1997). A Modern Approach to Probability Theory.

[29] Galichon, A., Ghoussoub, N.: Variational representations for N-cyclically monotone vector fields, (to appear in Pac. J. Math.)

[30] Gangbo, W., McCann, R.: Optimal maps in Monge’s mass transport problem. C. R. Acad. Sci. Paris. Ser. I. Math. **325**, 1653–1658 (1995)

[31] Gangbo W, McCann R (1996). The geometry of optimal transportation. Acta Math..

[32] Gangbo W, Swiech A (1998). Optimal maps for the multidimensional Monge–Kantorovich problem. Comm. Pure Appl. Math..

[33] Ghoussoub, N., Maurey, B.: Remarks on multi-marginal symmetric Monge–Kantorovich problems. (to appear in) Discret. Contin. Dyn. Syst. A (2013)

[34] Ghoussoub N, Moameni A (2013). A self-dual polar factorization for vector fields. Comm. Pure. Appl. Math..

[35] Ghoussoub, N., Moameni, A.: Symmetric Monge–Kantorovich problems and polar decompositions of vector fields, preprint (2013)

[36] Giraud BP, Peschanski R (2006). On positive functions with positive Fourier transforms. Acta Physica Polonica B.

[37] Hohenberg P, Kohn W (1964). Inhomogeneous electron gas. Phys. Rev. B.

[38] Heinich, H.: Probleme de Monge pour n probabilities. C. R. Math. Acad. Sci. Paris **334**(9), 793–795 (2002)

[39] Hu TC (2007). On pairwise independent and independent exchangeable random variables. Stoch. Anal. Appl..

[40] Ito, K.: An Introduction to Probability Theory. Cambridge University Press, Cambridge (1986)

[41] Janson S (1988). Some pairwise independent sequences for which the central limit theorem fails. Stochastics.

[42] Kim, Y.-H., Pass, B.: Multi-marginal optimal transport on Riemannian manifolds, preprint (2013)

[43] Katznelson, Y.: An Introduction to Harmonic Analysis. Cambridge University Press, Cambridge, New York, Melbourne (2004)

[44] Kerns JG, Szekely GJ (2006). De Finettis theorem for abstract finite exchangeable sequences. J. Theor. Probab..

[45] Kohn W, Sham LJ (1965). Self-consistent equations including exchange and correlation effects. Phys. Rev. A.

[46] Levy M (1979). Universal variational functionals of electron densities, first-order density matrices, and natural spin-orbitals and solution of the v-representability problem. Proc. Natl. Acad. Sci. USA.

[47] Lieb EH (1983). Density functionals for Coulomb systems. Int. J. Quantum Chem..

[48] Lieb EH, Oxford S (1981). Improved lower bound on the indirect Coulomb energy. Int. J. Quantum Chem..

[49] Pass B (2011). Uniqueness and Monge solutions in the multi-marginal optimal transportation problem. SIAM J. Math. Anal..

[50] Pass B (2012). On the local structure of optimal measures in the multi-marginal optimal transportation problem. Calc. Var. PDE.

[51] Pass B (2013). An upper bound on the semi-classical Hohenberg–Kohn functional. Nonlinearity.

[52] Pass B (2013). Optimal transportation with infinitely many marginals. J. Funct. Anal..

[53] Pass B (2013). On a class of optimal transportation problems with infinitely many marginals. SIAM J. Math. Anal..

[54] Pass B (2014). Multi-marginal optimal transport and multi-agent matching problems: uniqueness and structure of solutions. Discret. Contin. Dyn. Syst..

[55] Parr RG, Yang W (1995). Density-Functional Theory of Atoms and Molecules.

[56] Rappoport, D., Crawford, N.R.M., Furche, F., Burke, K.: Which density functional should I choose? In: Solomon, E.I., King, R.B., Scott, R.A. (eds.) Computational Inorganic and Bioinorganic Chemistry, Wiley, Chichester (2009)

[57] Räsänen E, Seidl M, Gori-Giorgi P (2011). Strictly correlated uniform electron droplets. Phys. Rev. B.

[58] Rudin, W.: Real and Complex Analysis, 3rd edn. McGraw Hill, Singapore (1987)

[59] Rüschendorf, L.: Bounds for distributions with multivariate marginals. In: Mosler, K., Scarsini, M. (eds.) Stochastic Orders and Decisions, IMS Lecture Notes vol. 19, pp. 285–310 (1991)

[60] Rüschendorf, L., Uckelmann, L.: On optimal multivariate couplings. In: Benes, V., Stepan, I. (eds.) Distributions with given marginals and moment problems. Kluwer, pp. 261–274 (1997)

[61] Seidl M (1999). Strong-interaction limit of density-functional theory. Phys. Rev. A.

[62] Seidl M, Gori-Giorgi P, Savin A (2007). Strictly correlated electrons in density-functional theory: a general formulation with applications to spherical densities. Phys. Rev. A.

[63] Seidl M, Perdew JP, Levy M (1999). Strictly correlated electrons in density-functional theory. Phys. Rev. A.

[64] Tao, T.: An Introduction to Measure Theory. http://terrytao.files.wordpress.com/2011/01/measure-book1

[65] Villani, C.: Optimal Transport. Springer, Berlin, Heidelberg (2009)

[66] Wendland, H.: Scattered Data Approximation. Cambridge University Press, Cambridge (2005)

